# Sex differences in the risk of retinopathy of prematurity: a systematic review, frequentist and Bayesian meta-analysis, and meta-regression

**DOI:** 10.1007/s12519-023-00775-x

**Published:** 2023-11-27

**Authors:** Tamara M. Hundscheid, Silvia Gulden, Mohamad F. Almutairi, František Bartoš, Giacomo Cavallaro, Eduardo Villamor

**Affiliations:** 1 Division of Neonatology, Department of Pediatrics, MosaKids Children’s Hospital, Maastricht University Medical Center (MUMC+), School for Oncology and Reproduction (GROW), Maastricht University, P. Debyelaan 25. P.O. Box 5800, 6202 AZ Maastricht, The Netherlands; 2 Neonatal Intensive Care Unit, Sant’Anna Hospital, Como, Italy; 3 Department of Psychology, University of Amsterdam, 1001 NK Amsterdam, The Netherlands; 4 Neonatal Intensive Care Unit, Fondazione IRCCS Ca’ Granda Ospedale Maggiore Policlinico, Milan, Italy

**Keywords:** Oxidative stress, Retinopathy of prematurity, Sex characteristics

## Abstract

**Background:**

Retinopathy of prematurity (ROP) is generally considered to be more frequent in males than in females. However, it is not known whether sex differences in ROP affect all degrees of the condition, are global and have changed as neonatology has developed. We aimed to conduct a systematic review and meta-analysis of studies addressing sex differences in the risk of developing ROP.

**Methods:**

PubMed/MEDLINE and Embase databases were searched. The frequentist, random-effects risk ratio (RR) and 95% confidence interval (CI) were calculated. Bayesian model averaged (BMA) meta-analysis was used to calculate the Bayes factors (BFs). The BF_10_ is the ratio of the probability of the data under the alternative hypothesis (H_1_) over the probability of the data under the null hypothesis (H_0_).

**Results:**

We included 205 studies (867,252 infants). Frequentist meta-analysis showed a positive association between male sex and severe ROP (113 studies, RR = 1.14, 95% CI = 1.07–1.22) but no association with any ROP (144 studies, RR = 1.00, 95% CI = 0.96–1.03). BMA showed extreme evidence in favor of H_1_ for severe ROP (BF_10_ = 71,174) and strong evidence in favor of H_0_ for any ROP (BF_10_ = 0.05). The association between male sex and severe ROP remained stable over time and was present only in cohorts from countries with a high or high-middle sociodemographic index.

**Conclusions:**

Our study confirms the presence of a male disadvantage in severe ROP but not in less severe forms of the disease. There are variations in the sex differences in ROP, depending on geographical location and sociodemographic level of the countries.

**Graphical abstract:**

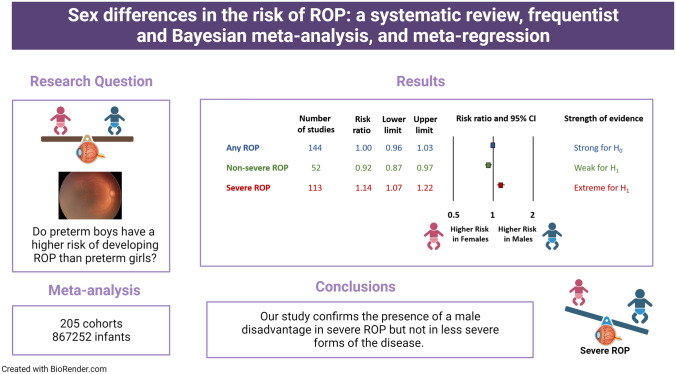

**Supplementary Information:**

The online version contains supplementary material available at 10.1007/s12519-023-00775-x.

## Introduction

Retinopathy of prematurity (ROP) is a major complication of preterm birth that can lead to varying degrees of visual impairment and even blindness [1, 2]. ROP is a multifactorial disease involving both intrinsic and environmental factors. Among the former, prematurity is the most important, with the rate of ROP being inversely proportional to infants' gestational age (GA) [1, 2]. Environmental factors include exposure to oxygen and/or oxidative stress, which play a key role in the pathogenesis of ROP. In addition, many other factors, such as genetic predisposition, perinatal infection/inflammation, or pre- and postnatal malnutrition, may also contribute to ROP [1, 2].

There is increasing evidence that sex differences exist in both the physiology and pathology of the eye [3–6]. In fact, sex hormones are locally produced in ocular tissues, and their receptors are present throughout the eye, including the retina [3, 4]. In addition, it is generally accepted that ROP is included in what is known as the "male disadvantage of prematurity" [7]. This concept is based on the notion that both mortality and morbidity associated with prematurity are higher in boys than in girls. However, sex differences in ROP have not been the subject of in-depth study until very recently.

In 2021, we performed a meta-analysis in which we included 41 studies that reported sex differences in various outcomes of prematurity, such as ROP, bronchopulmonary dysplasia, necrotizing enterocolitis, or intraventricular hemorrhage [7]. We found that severe ROP was more common in boys than in girls, but there were no sex differences for any ROP [7]. Very recently, Bahmani et al. conducted a meta-analysis of risk factors for any ROP and found no sex differences when they pooled the 121 studies that reported these data [8]. In addition, Hoyek et al. performed a meta-analysis of the male-to-female ratio in preterm infants requiring treatment for ROP [9]. They included 316 studies and found that a higher percentage of boys than girls were screened and treated for ROP [9]. These findings confirm the existence of a male disadvantage in the incidence of severe ROP. However, there are still questions to be answered.

Our aim in the present meta-analysis is to investigate sex differences not only in severe ROP but also in any ROP. In addition, we used subgroup analysis and meta-regression to determine whether the male disadvantage in ROP is homogeneous across geographic or sociodemographic areas and whether it has changed over time and with the subsequent evolution of neonatology. In addition to classical frequentist statistics, we used a Bayesian approach. By quantifying evidence on a continuous scale, Bayesian statistics allow more nuanced instead of all-or-none (significant vs. nonsignificant) conclusions [10, 11]. Furthermore, Bayesian meta-analysis can differentiate whether there is evidence for the alternative hypothesis (H_1_), for the null hypothesis (H_0_), or whether the data are inconclusive [10–12]. Therefore, Bayesian analysis can help distinguish between evidence of absence and absence of evidence.

## Methods

The methodology of this study is based on that of earlier studies by our group on sex differences and risk factors for outcomes of prematurity [7, 13–16]. The study was performed and reported according to the preferred reporting items for systematic reviews and meta-analyses and meta-analysis of observational studies in epidemiology guidelines. The review protocol was registered in the PROSPERO international register of systematic reviews (CRD42018095509). The research question was “Do preterm boys have a higher risk of developing ROP than preterm girls?”.

### Sources and search strategy

A comprehensive literature search was undertaken using the PubMed and Embase databases. The search strategy is detailed in Supplementary Table 1. No language limit was applied. The literature search was updated up to September 2022. Narrative reviews, systematic reviews, case reports, letters, editorials, and commentaries were excluded but were read to identify potential additional studies. Additional strategies to identify studies included manual review of reference lists from key articles that fulfilled our eligibility criteria, use of the “related articles” feature in PubMed, and use of the “cited by” tool in Web of Science and Google Scholar.

### Study selection and definitions

Studies were included if they had a prospective or retrospective cohort design, examined preterm infants (GA < 37 weeks) and reported primary data that could be used to measure the association between infant sex and rate of ROP. Studies that exclusively included late preterm infants (GA ≥ 34 weeks) or combined preterm and term infants were excluded. To identify relevant studies, two reviewers (HTM and GS) independently screened the results of the searches and applied inclusion criteria using a structured form. Discrepancies were resolved by two other reviewers (CG and VE).

Severe ROP was defined as prethreshold disease type 1 according to the Early Treatment for Retinopathy of Prematurity criteria (zone I, any stage ROP with plus disease; zone I, stage 3 ROP with or without plus disease; or zone II, stage 2 or 3 ROP with plus disease) [17] or as any ROP requiring treatment with photocryotherapy, laser photocoagulation or intravitreal injection of anti-vascular endothelial growth factor (VEGF) agents. Data on any ROP were also collected. If a study reported on any ROP and severe ROP, the non-severe ROP data were estimated by subtracting the severe ROP group from the any ROP group.

### Data extraction and assessment of study quality

Three investigators (HTM, GS and AMF) extracted data on the study design, demographics, and rate of ROP. A second group of investigators (GS and CG) checked the data extraction for completeness and accuracy. Methodological quality was assessed using the Newcastle‒Ottawa scale (NOS) for cohort studies [18]. This scale assigns a maximum of 9 points (4 for selection, 2 for comparability, and 3 for outcome). NOS scores ≥ 7 were considered high-quality studies (low risk of bias), and scores of 5 to 6 denoted moderate quality (moderate risk of bias) [18].

### Statistical analysis

#### Frequentist meta-analysis

Studies were combined and analyzed using Comprehensive meta-analysis V3.0 software (Biostat Inc., Englewood, NJ, USA) [19]. The risk ratio (RR) with 95% confidence interval (CI) was calculated for each individual study. Due to anticipated heterogeneity, summary statistics were calculated with a random-effects model. This model accounts for variability between studies as well as within studies [19, 20].

Statistical heterogeneity was assessed by Cochran’s *Q* statistic and by the *I*^2^ statistic. *I*^2^ was interpreted on the basis of Higgins and Thompson criteria, where 25%, 50%, and 75% correspond to low, moderate, and high heterogeneity, respectively [21]. Potential sources of heterogeneity were assessed through subgroup analysis and/or random effects (method of moments) univariate meta-regression analysis [22] as previously described [7, 13–15]. The potential sources of heterogeneity analyzed were exclusive inclusion of extremely preterm infants (GA ≤ 28 weeks), median year of cohort inclusion, median GA of the cohort, geographic location (continent or subcontinent), and sociodemographic index (SDI) quintile. The SDI is a composite measure of developmental status as it is associated with health outcomes, calculated as the geometric mean of the following three indicators: total rate of fertility, log income per capita, and mean years of education among those 15 years or older. SDI values are scaled from 0 (highest fertility, lowest income, and lowest education) to 1 (highest income, highest education, and lowest fertility) [23]. For comparisons across SDI quintiles, each country was assigned to a single quintile according to its SDI in 2019.

We used Egger’s regression test and funnel plots to assess publication bias. Duval and Tweedie's trim and fill method was used to adjust effect sizes in cases in which there was evidence of publication bias [24]. A probability value of less than 0.05 (0.10 for heterogeneity) was considered statistically significant.

### Bayesian model-averaged meta-analysis

The results were further supplemented by a Bayesian model-averaged meta-analysis (BMA) [10, 11]. BMA employs Bayes factors (BFs) and Bayesian model averaging to evaluate the likelihood of the data under the combination of models assuming the presence vs. the absence of the meta-analytic effect and heterogeneity [10, 11]. The BF_10_ is the ratio of the probability of the data under the alternative hypothesis (H_1_) over the probability of the data under the null hypothesis (H_0_). The BF_10_ was interpreted using the evidence categories suggested by Lee and Wagenmakers [25]. The evidence in favor of H_1_ (BF_10_ > 1) was categorized as weak/inconclusive (1 < BF_10_ < 3), moderate (3 < BF_10_ < 10), strong (10 < BF_10_ < 30), very strong (30 < BF_10_ < 100), and extreme (BF_10_ > 100). The evidence in favor of H_0_ (BF_10_ < 1) was categorized as weak/inconclusive (1/3 < BF_10_ < 1), moderate (1/10 < BF_10_ < 1/3), strong (1/30 < BF_10_ < 1/10), very strong (1/100 < BF_10_ < 1/30), and extreme (BF_10_ < 1/100). Consequently, BMA allows us to distinguish the absence of evidence from the evidence of absence [12]. The BF_rf_ is the ratio of the probability of the data under the random effects model over the probability of the data under the fixed effect model. The BF_rf_ was categorized in a similar way to that described for the BF_10_, considering values above 1 as evidence in favor of random effects (i.e., presence of heterogeneity) and values below 1 as evidence in favor of fixed effects (i.e., absence of heterogeneity). We used the empirical prior distributions based on the Cochrane Database of Systematics Reviews for logRR, logRR = Student’s *t* (*µ* = 0, *σ* = 0.32, *ν* = 3), tau = inverse-gamma (*k* = 1.51, *θ* = 0.23) [10, 11]. We performed BMA in JASP [26], which utilizes the metaBMA R package [27].

## Results

### Description of studies and quality assessment

The flow diagram of the search process is shown in Supplementary Fig. 1. Of 2320 potentially relevant studies, 205 (867,409 infants) were included. Their characteristics are summarized in Supplementary Table 2. The quality score of each study according to the NOS is depicted in Supplementary Table 2. All studies received a score of six or higher, indicating a low to moderate risk of bias.

### Main meta-analyses

**Fig. 1:**
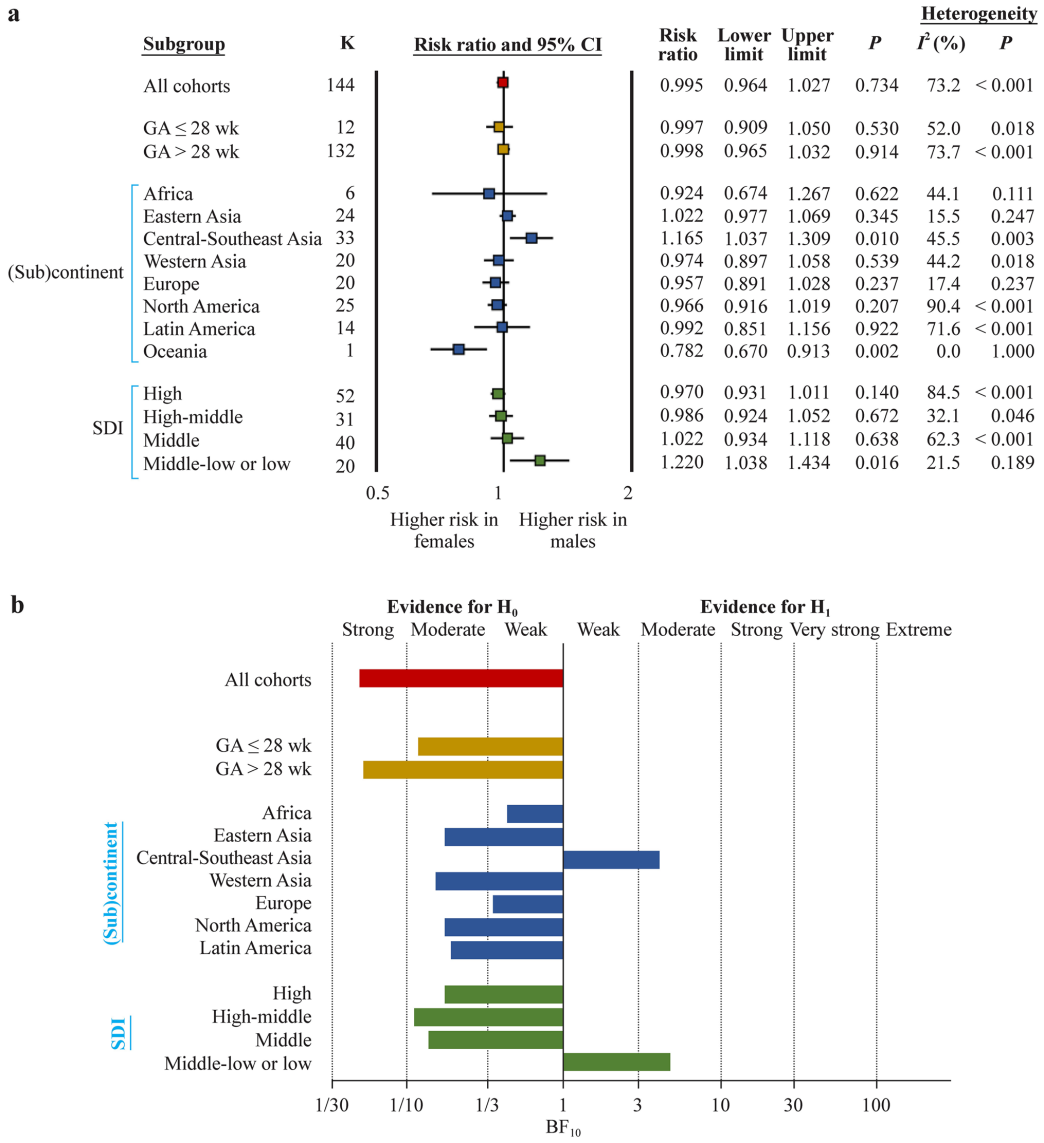
Any retinopathy of prematurity. **a** Summary of frequentist meta-analyses on the association between infant sex and any ROP. RR > 1 indicates a higher risk of ROP in males; **b** summary of Bayes factor (BF)_10_ values calculated through Bayesian model averaged meta-analysis. The BF_10_ is the ratio of the probability of the data under the alternative hypothesis (H_1_) over the probability of the data under the null hypothesis (H_0_). The BF_10_ was not calculated for Oceania because there was only one study from this continent. *ROP* retinopathy of prematurity, *RR* risk ratio, *CI* confidence interval, *SDI* sociodemographic index, *GA* gestational age

**Fig. 2:**
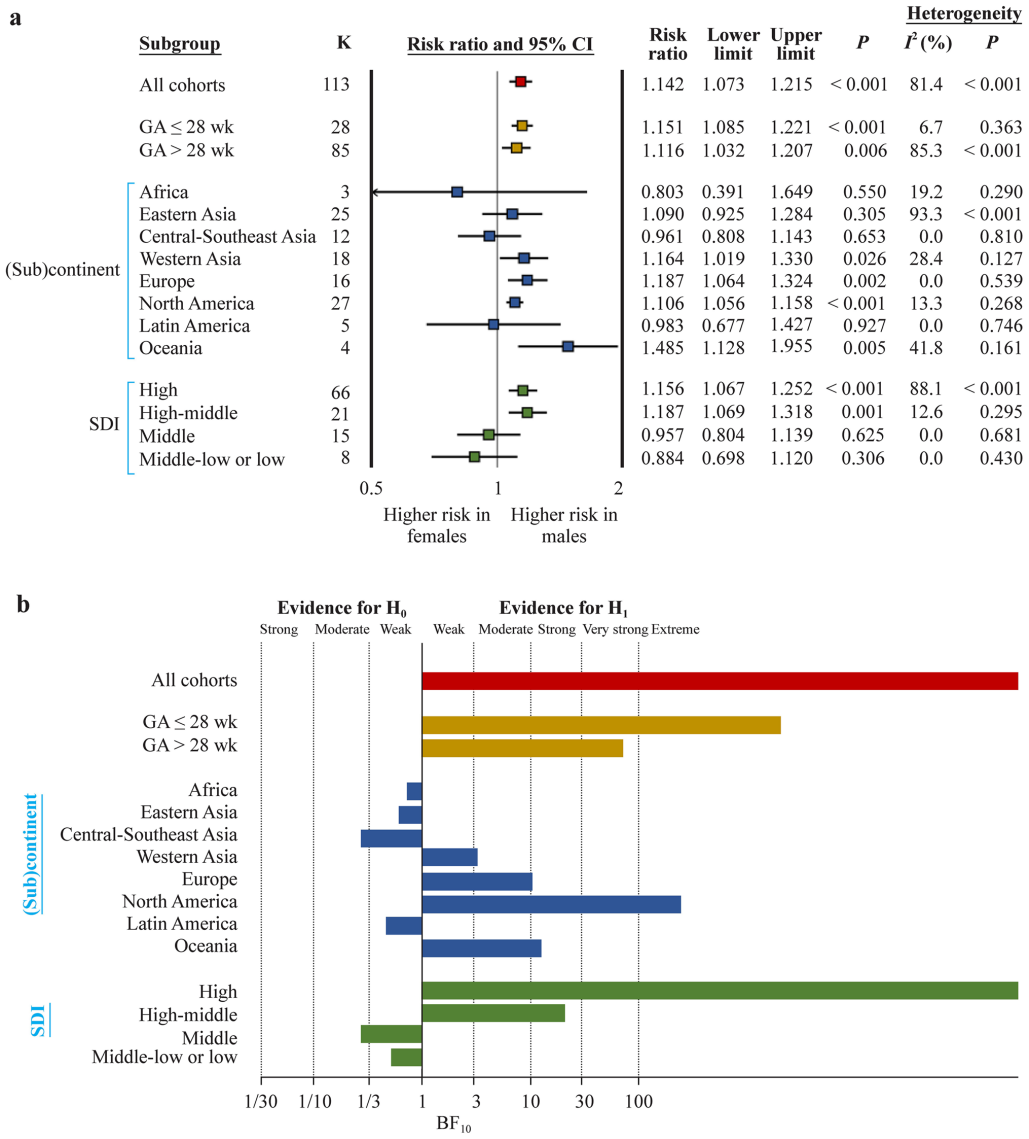
Severe retinopathy of prematurity. **a** Summary of frequentist meta-analyses on the association between infant sex and severe ROP. RR > 1 indicates a higher risk of ROP in males; **b** summary of Bayes factor (BF)_10_ values calculated through Bayesian model averaged meta-analysis. The BF_10_ is the ratio of the probability of the data under the alternative hypothesis (H_1_) over the probability of the data under the null hypothesis (H_0_). *ROP* retinopathy of prematurity, *RR* risk ratio, *CI* confidence interval, *SDI* sociodemographic index, *GA* gestational age

**Fig. 3:**
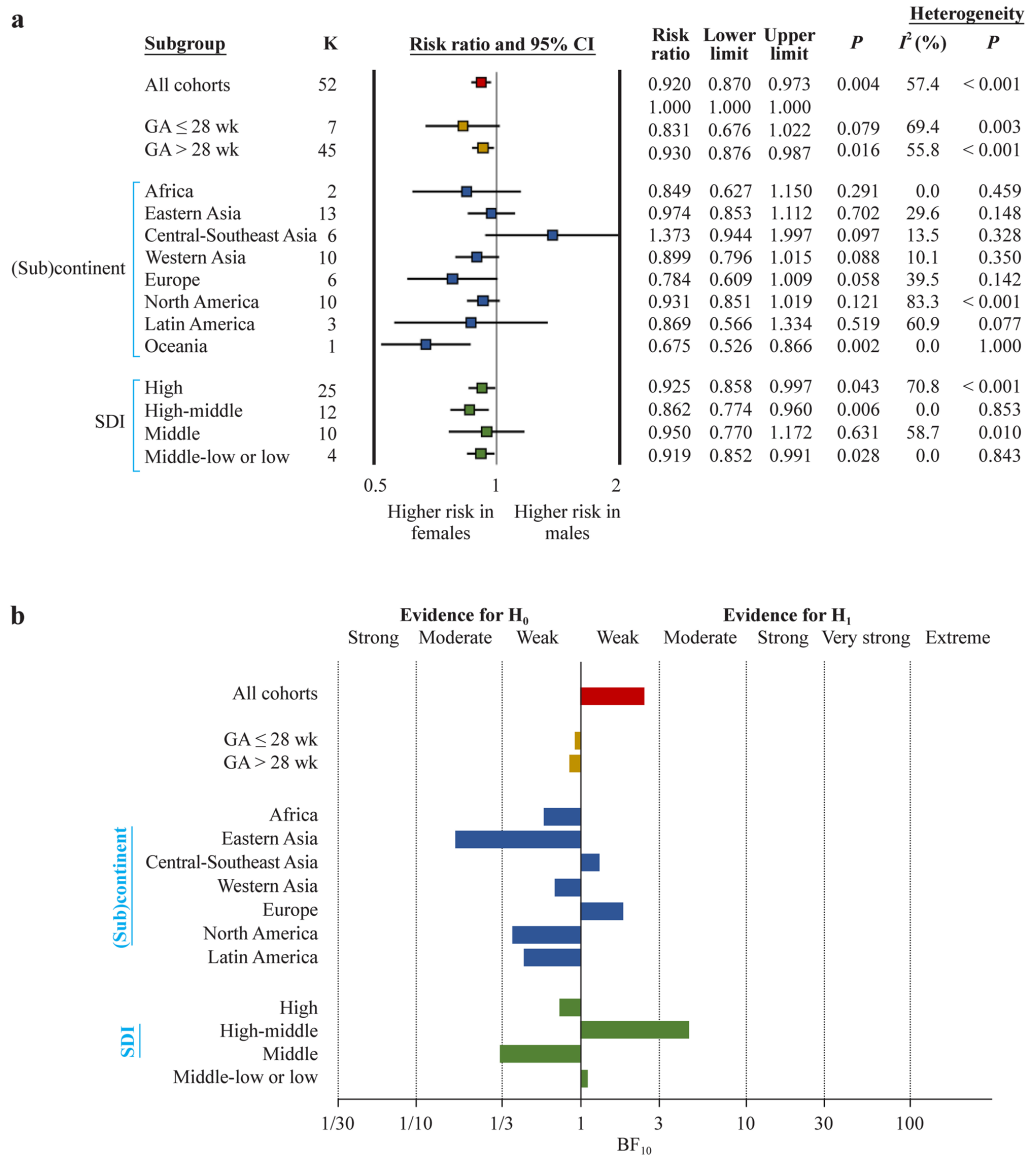
Non-severe retinopathy of prematurity. **a** Summary of frequentist meta-analyses on the association between infant sex and non-severe ROP. RR > 1 indicates a higher risk of ROP in males; **b** summary of Bayes factor (BF)_10_ values calculated through Bayesian model averaged meta-analysis. The BF_10_ is the ratio of the probability of the data under the alternative hypothesis (H_1_) over the probability of the data under the null hypothesis (H_0_). The BF_10_ was not calculated for Oceania because there was only one study from this continent. *ROP* retinopathy of prematurity, *RR* risk ratio, *CI* confidence interval, *SDI* sociodemographic index, *GA* gestational age

Frequentist meta-analysis did not show a significant association between sex and any ROP (144 studies, RR = 1.00, 95% CI = 0.96–1.03) (Fig. 1a) but did show a positive association between male sex and the risk of severe ROP (113 studies, RR = 1.14, 95% CI = 1.07–1.22) (Fig. 2a) and a negative association between male sex and the risk of non-severe ROP (52 studies, RR = 0.92, 95% CI = 0.87–0.97) (Fig. 3a).

BMA showed that the evidence in favor of H_0_ (i.e., lack of association between infant sex and ROP) was strong for any ROP (BF_10_ = 0.05) (Table 1, Fig. 1b), whereas the evidence in favor of H_1_ (i.e., infant sex is associated with ROP) was extreme for severe ROP (BF_10_ = 71,174) (Table 2, Fig. 2b) and weak for non-severe ROP (BF_10_ = 2.42) (Table 3, Fig. 3b).

**Table 1:** Bayesian model averaged meta-analysis of the association between infant sex and any retinopathy of prematurity

Variables	Groups	*K*	Averaged effect (logRR)	Standard deviation	Credible interval		BF_10_	Evidence for		*P* frequentist analysis	BF_rf_	Evidence for	
					Lower limit	Upper limit		H_1_	H_0_			Random effects	Fixed effects
GA	All	144	− 0.004	0.017	− 0.037	0.031	0.050		Strong	0.734	> 10^12^	Extreme	
	≤ 28 wk	12	− 0.021	0.044	− 0.116	0.059	0.118		Moderate	0.530	2.183	Weak	
	> 28 wk	132	0.000	0.019	− 0.035	0.037	0.052		Strong	0.914	> 10^12^	Extreme	
(Sub)continent	Africa	6	− 0.083	0.115	− 0.306	0.151	0.437		Weak	0.622	0.835		Weak
	Eastern Asia	24	0.025	0.023	− 0.028	0.064	0.178		Moderate	0.345	0.534		Weak
	Central-Southeast Asia	33	0.141	0.057	0.031	0.256	4.100	Moderate		0.010	71.972	Very strong	
	Western Asia	20	− 0.026	0.043	− 0.107	0.062	0.154		Moderate	0.539	548.2	Extreme	
	Europe	20	− 0.051	0.035	− 0.116	0.022	0.349		Weak	0.237	0.518		Weak
	North America	25	− 0.035	0.030	− 0.096	0.025	0.172		Moderate	0.207	> 10^12^	Extreme	
	Latin America	14	− 0.001	0.069	− 0.138	0.133	0.193		Moderate	0.922	> 10^5^	Extreme	
SDI	High	52	− 0.031	0.021	− 0.072	0.011	0.176		Moderate	0.140	> 10^12^	Extreme	
	High-middle	31	− 0.012	0.036	− 0.080	0.061	0.110		Moderate	0.672	266.4	Extreme	
	MiddIe	40	0.021	0.044	− 0.065	0.108	0.138		Moderate	0.638	> 10^6^	Extreme	
	Middle-low/low	20	0.184	0.074	0.039	0.330	4.762	Moderate		0.016	0.974		Weak

Number of studies per country/territory: Africa: Egypt 2, Ghana, Nigeria 2, Rwanda; Eastern Asia: China 10, Japan 2, Korea 6, Taiwan Province of China 6; Western Asia: Israel, Kuwait, Oman, Palestine, Saudi Arabia 2, Turkey 14; Central Southeast Asia: Bangladesh 2, Brunei, India 11, Indonesia 2, Iran 13, Malaysia, Pakistan 2, Thailand; Europe: Croatia, Denmark, Germany 3, Greece, Italy 2, Portugal, Serbia 2, Slovenia, Spain 4, Sweden 3, The Netherlands; North America: Canada, USA 24; Latin America: Brazil 5, Chile, Colombia, Cuba, Mexico 4, Peru 2; Oceania: Australia. High SDI: Australia, Brunei, Canada, Denmark, Germany 3, Japan 2, Korea 6, Kuwait, Slovenia, Sweden 3, Sweden/USA/Germany, Taiwan Province of China 6, Thailand, The Netherlands, USA 24; high-middle SDI: Chile, Croatia, Greece, Israel, Italy 2, Malaysia, Oman, Portugal, Saudi Arabia 2, Serbia 2, Spain 4, Turkey 14; middle SDI: Brazil 5, China 10, Colombia, Cuba, Egypt 2, Indonesia 2, Iran 13, Mexico 5, Peru 2; middle-low to low: Bangladesh 2, Ghana, India 11, Nigeria 2, Pakistan 2, Palestine, Rwanda. *RR* risk ratio, *GA* gestational age, *BF* Bayes factor, *SDI* sociodemographic index

**Table 2:** Bayesian model averaged meta-analysis of the association between infant sex and severe retinopathy of prematurity

Variables	Groups	*K*	Averaged effect (logRR)	Standard deviation	Credible interval		BF_10_	Evidence for		*P* frequentist analysis	BF_rf_	Evidence for	
					Lower limit	Upper limit		H_1_	H_0_			Random effects	Fixed effects
GA	All	113	0.132	0.023	0.086	0.178	71,175	Extreme		< 0.001	> 10^12^	Extreme	
	≤ 28 wk	28	0.134	0.030	0.079	0.196	2030	Extreme		< 0.001	0.188		Moderate
	> 28 wk	85	0.115	0.030	0.057	0.173	73.05	Very strong		0.006	> 10^12^	Extreme	
(Sub)continent	Africa	3	− 0.106	0.247	− 0.617	0.362	0.723		Weak	0.550	0.859		Weak
	Eastern Asia	25	0.093	0.060	− 0.030	0.207	0.622		Weak	0.305	> 10^12^	Extreme	
	Central-Southeast Asia	12	− 0.037	0.089	− 0.212	0.136	0.275		Moderate	0.653	0.350		Weak
	Western Asia	18	0.134	0.057	0.026	0.251	3.213	Moderate		0.026	0.978		Weak
	Europe	16	0.170	0.060	0.055	0.293	10.48	Strong		0.002	0.473		Weak
	North America	27	0.079	0.029	0.040	0.152	242.8	Extreme		< 0.001	0.409		Weak
	Latin America	5	− 0.012	0.168	− 0.345	0.318	0.472		Weak	0.927	0.652		Weak
	Oceania	4	0.325	0.119	0.089	0.568	12.72	Strong		0.005	1.028	Weak	
SDI	High	69	0.142	0.027	0.089	0.196	10,335	Extreme		< 0.001	> 10^12^	Extreme	
	High-middle	21	0.157	0.054	0.059	0.272	21.17	Strong		0.001	0.658	Extreme	
	MiddIe	15	− 0.030	0.092	− 0.208	0.153	0.278		Moderate	0.625	0.609	Extreme	
	Middle-low to low	8	− 0.113	0.122	− 0.358	0.122	0.526		Weak	0.306	0.572		Weak

Number of studies per country/territory: Africa: Egypt, Rwanda, South Africa; Eastern Asia: China, Japan 6, Korea 8, Taiwan Province of China 6; Western Asia: Israel, Kuwait 2, Saudi Arabia, Turkey 15; Central-Southeast Asia: Bangladesh, India 6, Indonesia, Iran 2, Malaysia, Singapore; Europe: Austria, Croatia, Denmark, Germany 2, Serbia, Slovenia, Spain 2, Sweden 6, UK; North America: Canada, USA 25, USA/Canada; Latin America: Brazil 3, Mexico, Peru; Oceania: Australia 3, Australia/New Zealand. High SDI: Australia 3, Australia/New Zealand, Austria, Canada, Denmark, Germany 2, Japan 6, Korea 8, Kuwait 2, Singapore, Slovenia, Sweden 6, Sweden/USA/Germany, Sweden/USA, Taiwan Province of China 6, UK, USA 25, USA/Canada; high-middle SDI: Croatia, Israel, Malaysia, Saudi Arabia, Serbia, Spain 2, Turkey 15; middle SDI: Brazil 3, China 5, Egypt, Indonesia, Iran 2, Mexico, Peru, South Africa; middle-low to low: Bangladesh, India 6, Rwanda. *RR* risk ratio, *GA* gestational age, *BF* Bayes factor, *SDI* sociodemographic index

**Table 3:** Bayesian model averaged meta-analysis of the association between infant sex and non-severe retinopathy of prematurity

Variables	Groups	*K*	Averaged effect (logRR)	Standard deviation	Credible interval		BF_10_	Evidence for		*P* value frequentist analysis	BF_rf_	Evidence for	
					Lower limit	Upper limit		H_1_	H_0_			Random effects	Fixed effects
GA	All	52	− 0.085	0.033	− 0.152	− 0.020	2.418	Weak		0.004	688,892	Extreme	
	≤ 28 wk	7	− 0.149	0.107	− 0.379	0.049	0.928		Weak	0.079	7.125	Moderate	
	> 28 wk	45	− 0.071	0.035	− 0.141	− 0.002	0.842		Weak	0.016	126,974	Extreme	
(Sub)continent	Africa	2	− 0.113	0.162	− 0.428	0.212	0.606		Weak	0.702	0.777		Weak
	Eastern Asia	13	− 0.020	0.061	− 0.145	0.099	0.175		Moderate	0.097	0.856		Weak
	Central-Southeast Asia	6	0.231	0.168	− 0.092	0.570	1.285	Weak		0.088	1.105	Weak	
	Western Asia	10	− 0.102	0.065	− 0.229	0.025	0.706		Weak	0.058	0.678		Weak
	Europe	6	− 0.188	0.103	− 0.402	0.005	1.802	Weak		0.121	0.920		Weak
	North America	10	− 0.075	0.068	− 0.212	0.059	0.383		Weak	0.519	45,277	Extreme	
	Latin America	3	− 0.082	0.145	− 0.367	0.205	0.451		Weak	0.002	1.105	Weak	
SDI	High	26	− 0.083	0.046	− 0.176	0.006	0.739		Weak	0.043	300,479	Extreme	
	High-middle	12	− 0.144	0.056	− 0.254	− 0.035	4.475	Moderate		0.006	0.280		Moderate
	MiddIe	10	− 0.051	0.097	− 0.238	0.149	0.325		Moderate	0.631	5.856	Moderate	
	Middle-low/low	4	0.240	0.228	− 0.182	0.716	1.086	Weak		0.028	0.776		Weak

Number of studies per country/territory: Africa: Egypt, Rwanda; Eastern Asia: China 3, Japan 2, Korea 5, Taiwan Province of China 3; Western Asia: Kuwait, Saudi Arabia, Turkey 8; Central-Southeast Asia: Bangladesh, India 2, Indonesia, Iran 2; Europe: Croatia, Germany, Serbia, Slovenia, Spain, Sweden; North America: USA 10; Latin America: Brazil 3; Oceania: Australia. High SDI: Australia, Germany, Japan 2, Korea 5, Kuwait, Slovenia, Sweden, Sweden/USA/Germany, Taiwan Province of China 3, USA 10; high-middle SDI: Croatia, Saudi Arabia, Serbia, Spain, Turkey 8; middle SDI: Brazil 3, China 3, Egypt, Indonesia, Iran 2; middle-low to low: Bangladesh, India 2, Rwanda. *RR* risk ratio, *GA* gestational age, *BF* Bayes factor, *SDI* sociodemographic index

The heterogeneity of the three meta-analyses was very high: any ROP: *I*^2^ = 73.2%, BF_rf_ > 10^6^ (extreme evidence for random effects); severe ROP: *I*^2^ = 81.4%, BF_rf_ > 10^6^ (extreme evidence for random effects); non-severe ROP: *I*^2^ = 57.4%, BF_rf_ > 10^5^ (extreme evidence for random effects). Detailed data on the heterogeneity of BMA analyses are depicted in Supplementary Tables 3, 4, and 5. Visual inspection of the funnel plot, Duval and Tweedie's trim and fill procedure (number of imputed studies was zero), and Egger's test of the intercept did not indicate significant publication bias in the meta-analyses of severe and non-severe ROP (Supplementary Fig. 2). For the meta-analysis on any ROP, Duvall and Tweedie's trim and fill procedure suggested that 36 studies were missing and that the adjusted RR was 0.94 (95% CI = 0.91–0.97).

### Subgroup analysis and meta-regression

Subgroup analysis based on the exclusive inclusion of extremely preterm infants (GA ≤ 28 weeks) did not reveal any substantial differences for any ROP (Fig. 1, Table 1), severe ROP (Fig. 2, Table 2), or non-severe ROP (Fig. 3, Table 3).

Subgroup analysis based on the geographic location of the cohorts showed a frequentist positive association between male sex and any ROP in the cohorts from Central-Southeast Asia (RR = 1.17, 95% CI = 1.04–1.31), and the BMA showed that the evidence in favor of H_1_ was moderate for this association (BF_10_ = 4.1) (Fig. 1, Table 1). For Oceania (one study), there was a significantly higher risk of any ROP for female infants than for male infants (Fig. 1a). In all other geographic locations, no evidence of an association between sex and any ROP was found. The frequentist positive association between male sex and severe ROP was significant for North America, Europe, Oceania, and Western Asia but not for the other (sub)continents (Fig. 2). BMA showed that the evidence in favor of H_1_ (association between male sex and severe ROP) was extreme for North America, strong for Europe and Oceania, and moderate for Western Asia (Fig. 2, Table 2). In contrast, the evidence in favor of H_0_ (lack of association between sex and severe ROP) was moderate for Central–Southeast Asia and weak for Africa, Eastern Asia and Latin America (Fig. 2, Table 2). The negative association between male sex and non-severe ROP was observed only in the frequentist meta-analysis pooling all the studies but was not significant for any (sub)continent (Fig. 3a). BMA showed that the evidence for H_1_ (association between infant sex and non-severe ROP) was weak for Central-Southeast Asia and Europe, and the evidence for H_0_ was moderate for Eastern Asia and weak for Africa, Western Asia, North America, and Latin America (Fig. 3, Table 3).

Subgroup analysis based on SDI showed a significant positive association between any ROP and male sex in the middle-low to low SDI subgroup (RR = 1.22, 95% CI = 1.04–1.43), and BMA showed that the evidence in favor of this association was moderate (BF_10_ = 4.8) (Fig. 1, Table 1). The frequentist positive association between male sex and severe ROP was significant for the cohorts from the high and high-middle SDI countries (Fig. 2). BMA showed that the evidence in favor of H_1_ (association between infant sex and severe ROP) was extreme for the high SDI cohorts (BF_10_ = 71,174) and strong (BF_10_ = 21.2) for the high-middle SDI cohorts (Fig. 2, Table 2). In contrast, the evidence in favor of H_0_ (lack of association between infant sex and severe ROP) was moderate for the middle SDI cohorts (BF_10_ = 0.28) (Fig. 2, Table 2). Regarding non-severe ROP, an association with sex was observed in the frequentist meta-analysis of the high, high-middle, and middle to low SDI subgroups (Fig. 3). BMA showed that the evidence in favor of H_1_ (association non-severe ROP and sex) was moderate for the high-middle SDI cohorts (BF_10_ = 4.5), whereas the evidence in favor of H_0_ was moderate for the middle SDI cohorts (BF_10_ = 0.28).

Meta-regression showed that the effect size of the association between sex and ROP remained stable over time. That is, it does not correlate with the median year of birth of the cohort (Supplementary Table 6, Supplementary Fig. 3). This lack of correlation was maintained after the exclusion of studies with a time span of more than 5 years (Supplementary Table 6, Supplementary Fig. 3). The meta-regression could also not demonstrate a correlation between the mean/median GA of the cohort and the effect size of the association between sex and ROP (Supplementary Table 6).

## Discussion

To our knowledge, this is the most comprehensive meta-analysis to date on sex differences in the development of ROP. Our data confirm the previous observations that the more severe forms of ROP are more common in preterm boys than in preterm girls [9]. However, when the study focused on any ROP, we did not observe sex differences. Therefore, our data suggest that male sex might be associated with a greater ROP progression rate. In addition, subgroup analysis showed wide variation in the association between sex and ROP according to geographic location and sociodemographic level of the countries. Finally, meta-regression showed that sex differences in ROP have remained stable over the years.

In the presence of an immature antioxidant system, hyperoxia and oxidative stress play a key role in ROP pathogenesis [1, 28]. Therefore, sex differences in ROP incidence may be related to sex differences in the retinal response to oxygen. Retinal vascular development occurs while the fetus is in the uterus in a relatively hypoxic environment. When an infant is delivered very prematurely, retinal development must continue in a hyperoxic environment, even in room air [1]. In addition, preterm infants frequently need supplementary oxygen. In the first phase, hyperoxia induces the suppression of angiogenic factors, such as VEGF, leading to cessation of vascularization and loss of normal vessels. This results in hypoxia in the avascular retina, which induces an increase in vascular growth factors, leading to the formation of abnormal vessels in phase 2 of ROP (from 30 to 32 weeks postmenstrual age to term) [1].

The antioxidant defense system develops more rapidly in female fetuses than in males, and this difference, particularly in the glutathione pathway, is often suggested as a key factor in the male disadvantage of prematurity [29, 30]. Moreover, the increased female resistance to oxidative stress may even be present at the cellular level. For example, mitochondria from female cells generate fewer superoxide radicals than those from males [31], and cell cultures of female endothelial cells from umbilical veins [32] or fetal pulmonary arteries [33] are more resistant to hyperoxia-induced damage than male cells. This enhanced female antioxidant capacity may confer greater protection against ROP during the hyperoxic phase and is a potential explanation for the sex differences we found. Accordingly, it is noteworthy that in randomized controlled trials comparing a lower oxygen saturation target (85–89%) with a higher saturation target (91%–95%), lower saturation levels protected against severe ROP only in preterm boys but not in girls [34].

It should be noted that ROP is a multifactorial condition, and therefore, many of the potential pre- and postnatal risk factors may differ by sex. Fetal sex is an important influence on prenatal development, not only because of genetic differences between males and females but also because of differences in sex hormones [35–37]. The levels of sex hormones diverge very early between male and female embryos/fetuses [35, 36], and this difference is maintained in early postnatal life [38]. This differential concentration of sex hormones may result in variations in the growth and development of almost all organs and systems [29, 39, 40], including the retina [41]. Within these sex-specific developmental trajectories, that of the lung is particularly relevant [29, 39, 40]. Slower lung maturation among male fetuses may result in a more challenging clinical condition at birth, which is of great importance for the subsequent morbidity and mortality of very preterm infants [29, 39, 40]. In our previous meta-analysis, we observed that male sex in very preterm infants was associated with higher rates of intubation at birth, respiratory distress syndrome treated with surfactant and/or mechanical ventilation, pneumothorax, sepsis, and necrotizing enterocolitis [7]. These differences in clinical course and complications may have a significant impact on the progression of ROP, as male preterm infants are more often exposed to inflammation, higher oxygen levels, and oxidative stress.

An important limitation of our meta-analysis is the high degree of heterogeneity. When we examined this heterogeneity by subgroup analysis, we found large variations in sex differences in ROP according to geographical location and sociodemographic level. The global epidemiological trend of ROP over the last decades has been variable, depending on the socioeconomic and demographic conditions of the countries [42–44]. In high-income countries, ROP mainly affects extremely preterm infants, and ROP screening is not routinely recommended for infants born after 30 weeks’ GA and with birth weights greater than 1500 g [45, 46]. In low- and middle-income countries, the incidence of preterm birth is increasing, while the mortality rate of preterm babies is decreasing as neonatal care units are improving in number and capability. However, primary prevention of ROP through close oxygen monitoring and titration can be challenging due to limited human and material resources [42–44, 46]. Therefore, low- and middle-income nations are currently experiencing epidemic levels of ROP not only in extremely premature infants but also in larger, more mature infants [42–44, 46]. Our data suggest that sex differences in ROP vary between these two epidemiological patterns of the disease.

It should be noted that most of the cohorts were from countries with high economic and educational levels but low fertility rates (i.e., high and high-middle SDI quintiles). The results from these countries account for the extreme evidence we found in favor of male disadvantage in severe ROP. However, there is a marked difference with the results from countries with medium and low SDIs. For example, there was no evidence of sex differences in severe ROP in the lower SDI groups. However, a male disadvantage was observed for any ROP, which was not present in the meta-analysis of high and medium SDI countries.

Finally, it has been suggested that advances in perinatal medicine, which have led to reductions in mortality and improvements in short-term outcomes for the most vulnerable preterm infants, may have had a greater impact on boys than on girls [47]. It is, therefore, possible that the male disadvantage of prematurity diminishes over the years. This seems to be the case with mortality but not with other complications of prematurity [7]. Our meta-analysis included cohorts from the last 40 years, and meta-regression confirmed that sex differences in ROP have remained stable over time.

There is a growing awareness among clinicians and researchers that many of the diseases they treat and study are characterized by sex/gender differences in epidemiology, pathophysiology, clinical manifestations, disease progression, and response to treatment [3–6, 30]. The present meta-analysis confirms that the risk of developing severe ROP is higher in preterm boys than in girls. Visual impairment is an important sequela of prematurity that affects both the anterior and posterior visual pathways [48]. Some conditions leading to posterior visual pathway dysfunction, such as intraventricular hemorrhage, posthemorrhagic ventricular dilation, or periventricular leukomalacia, are also included in the male disadvantage of prematurity [7]. This raises the risk of visual dysfunction in extremely premature males. An increased risk of visual impairment may play a role in extending the male disadvantage of prematurity beyond the first years of life, as evidenced by the greater risk of neurodevelopmental impairment in ex-preterm boys compared with ex-preterm girls [49, 50].

### Supplementary Information

Below is the link to the electronic supplementary material.

Supplementary file 1 (PDF 1500 KB)

## Data Availability

All data relevant to the study are included in the article or uploaded as supplementary information. Additional data are available upon reasonable request.

## References

[1] Cavallaro G, Filippi L, Bagnoli P, La Marca G, Cristofori G, Raffaeli G (2014). The pathophysiology of retinopathy of prematurity: an update of previous and recent knowledge. Acta Ophthalmol.

[2] Hellström A, Smith LEH, Dammann O (2013). Retinopathy of prematurity. Lancet.

[3] Nuzzi R, Scalabrin S, Becco A, Panzica G (2018). Gonadal hormones and retinal disorders: a review. Front Endocrinol (Lausanne).

[4] Cascio C, Deidda I, Russo D, Guarneri P (2015). The estrogenic retina: the potential contribution to healthy aging and age-related neurodegenerative diseases of the retina. Steroids.

[5] Clayton JA, Davis AF (2015). Sex/gender disparities and women's eye health. Curr Eye Res.

[6] Aninye IO, Digre K, Hartnett ME, Baldonado K, Shriver EM, Periman LM (2021). The roles of sex and gender in women's eye health disparities in the United States. Biol Sex Differ.

[7] van Westering-Kroon E, Huizing MJ, Villamor-Martínez E, Villamor E (2021). Male disadvantage in oxidative stress-associated complications of prematurity: a systematic review, meta-analysis and meta-regression. Antioxidants (Basel).

[8] Bahmani T, Karimi A, Rezaei N, Daliri S (2022). Retinopathy prematurity: a systematic review and meta-analysis study based on neonatal and maternal risk factors. J Matern Fetal Neonatal Med.

[9] Hoyek S, Peacker BL, Acaba-Berrocal LA, Al-Khersan H, Zhao Y, Hartnett ME (2022). The male to female ratio in treatment-warranted retinopathy of prematurity: a systematic review and meta-analysis. JAMA Ophthalmol.

[10] Bartoš F, Otte WM, Gronau QF, Timmers B, Otte WM, Ly A, et al. Empirical prior distributions for Bayesian meta-analyses of binary and time to event outcomes. 2023. arXiv. 2306.11468.

[11] Bartoš F, Gronau QF, Timmers B, Otte WM, Ly A, Wagenmakers EJ (2021). Bayesian model-averaged meta-analysis in medicine. Stat Med.

[12] Altman DG, Bland JM (1995). Statistics notes: absence of evidence is not evidence of absence. BMJ.

[13] Borges-Lujan M, Gonzalez-Luis GE, Roosen T, Huizing MJ, Villamor E (2022). Sex differences in patent ductus arteriosus incidence and response to pharmacological treatment in preterm infants: a systematic review, meta-analysis and meta-regression. J Pers Med.

[14] Hundscheid TM, Huizing MJ, Villamor-Martinez E, Bartoš F, Villamor E (2023). Association of funisitis with short-term outcomes of prematurity: a frequentist and bayesian meta-analysis. Antioxidants (Basel).

[15] Villamor-Martinez E, Cavallaro G, Raffaeli G, Mohammed Rahim OMM, Gulden S, Ghazi AM (2018). Chorioamnionitis as a risk factor for retinopathy of prematurity: an updated systematic review and meta-analysis. PLoS ONE.

[16] Cavallaro G, Villamor-Martinez E, Filippi L, Mosca F, Villamor E (2017). Probiotic supplementation in preterm infants does not affect the risk of retinopathy of prematurity: a meta-analysis of randomized controlled trials. Sci Rep.

[17] Good WV, Hardy RJ, Dobson V, Palmer EA, Phelps DL, Early Treatment for Retinopathy of Prematurity Cooperative Group (2010). Final visual acuity results in the early treatment for retinopathy of prematurity study. Arch Ophthalmol.

[18] Stang A (2010). Critical evaluation of the Newcastle–Ottawa scale for the assessment of the quality of nonrandomized studies in meta-analyses. Eur J Epidemiol.

[19] Borenstein M, Egger M, Higgins JPT, Smith GD (2022). Comprehensive meta-analysis software. Systematic reviews in health research: meta-analysis in context.

[20] Borenstein M, Higgins J (2013). Meta-analysis and subgroups. Prev Sci.

[21] Higgins JP, Thompson SG (2002). Quantifying heterogeneity in a meta-analysis. Stat Med.

[22] Borenstein M, Hedges LV, Higgins JP, Rothstein HR (2021). Introduction to meta-analysis.

[23] Kassebaum N, Kyu HH, Zoeckler L, Olsen HE, Thomas K, Global Burden of Disease Child and Adolescent Health Collaboration (2017). Child and adolescent health from 1990 to 2015: findings from the global burden of diseases, injuries, and risk factors 2015 study. JAMA Pediatr.

[24] Duval S, Tweedie R (2000). Trim and fill: a simple funnel-plot-based method of testing and adjusting for publication bias in meta-analysis. Biometrics.

[25] Lee M, Wagenmakers EJ (2013). Bayesian data analysis for cognitive science: a practical course.

[26] JASP Team. Computer software (version 0.17.3). 2023.

[27] Heck D, Gronau F, Wagenmakers E. metaBMA: Bayesian model averaging for random and fixed effects meta-analysis (R package version 0.6.1). 2019. https://CRAN.R-project.org/package=metaBMA.

[28] Stone WL, Shah D, Hollinger SM (2016). Retinopathy of prematurity: an oxidative stress neonatal disease. Front Biosci (Landmark Ed).

[29] Lorente-Pozo S, Parra-Llorca A, Torres B, Torres-Cuevas I, Nuñez-Ramiro A, Cernada M (2018). Influence of sex on gestational complications, fetal-to-neonatal transition, and postnatal adaptation. Front Pediatr.

[30] Lavoie JC, Tremblay A (2018). Sex-specificity of oxidative stress in newborns leading to a personalized antioxidant nutritive strategy. Antioxidants (Basel).

[31] Diaz-Castro J, Pulido-Moran M, Moreno-Fernandez J, Kajarabille N, de Paco C, Garrido-Sanchez M (2016). Gender specific differences in oxidative stress and inflammatory signaling in healthy term neonates and their mothers. Pediatr Res.

[32] Zhang Y, Lingappan K (2017). Differential sex-specific effects of oxygen toxicity in human umbilical vein endothelial cells. Biochem Biophys Res Commun.

[33] Zhang Y, Dong X, Shirazi J, Gleghorn JP, Lingappan K (2018). Pulmonary endothelial cells exhibit sexual dimorphism in their response to hyperoxia. Am J Physiol Heart Circ Physiol.

[34] Askie LM, Darlow BA, Finer N, Schmidt B, Stenson B, Tarnow-Mordi W (2018). Association between oxygen saturation targeting and death or disability in extremely preterm infants in the neonatal oxygenation prospective meta-analysis collaboration. JAMA.

[35] Knickmeyer RC, Baron-Cohen S (2006). Fetal testosterone and sex differences. Early Hum Dev.

[36] Ainsworth C (2015). Sex redefined. Nature.

[37] Rosenfeld CS (2015). Sex-specific placental responses in fetal development. Endocrinology.

[38] Greaves RF, Pitkin J, Ho CS, Baglin J, Hunt RW, Zacharin MR (2015). Hormone modeling in preterm neonates: establishment of pituitary and steroid hormone reference intervals. J Clin Endocrinol Metab.

[39] Raghavan D, Jain R (2016). Increasing awareness of sex differences in airway diseases. Respirology.

[40] Seaborn T, Simard M, Provost PR, Piedboeuf B, Tremblay Y (2010). Sex hormone metabolism in lung development and maturation. Trends Endocrinol Metab.

[41] Salyer DL, Lund TD, Fleming DE, Lephart ED, Horvath TL (2001). Sexual dimorphism and aromatase in the rat retina. Brain Res Dev Brain Res.

[42] Sabri K, Ells AL, Lee EY, Dutta S, Vinekar A (2022). Retinopathy of prematurity: a global perspective and recent developments. Pediatrics.

[43] Bowe T, Nyamai L, Ademola-Popoola D, Amphornphruet A, Anzures R, Cernichiaro-Espinosa LA (2019). The current state of retinopathy of prematurity in India, Kenya, Mexico, Nigeria, Philippines, Romania, Thailand, and Venezuela. Digit J Ophthalmol.

[44] Coyner AS, Oh MA, Shah PK, Singh P, Ostmo S, Valikodath NG (2022). External validation of a retinopathy of prematurity screening model using artificial intelligence in 3 low-and middle-income populations. JAMA Ophthalmol.

[45] Prakalapakorn SG, Greenberg L, Edwards EM, Ehret DE (2021). Trends in retinopathy of prematurity screening and treatment: 2008–2018. Pediatrics.

[46] Gilbert C, Fielder A, Gordillo L, Quinn G, Semiglia R, Visintin P (2005). Characteristics of infants with severe retinopathy of prematurity in countries with low, moderate, and high levels of development: implications for screening programs. Pediatrics.

[47] Garfinkle J, Yoon EW, Alvaro R, Nwaesei C, Claveau M, Lee SK (2020). Trends in sex-specific differences in outcomes in extreme preterms: progress or natural barriers?. Arch Dis Child Fetal Neonatal Ed.

[48] Siatkowski RM, Good WV, Summers CG, Quinn GE, Tung B (2013). Clinical characteristics of children with severe visual impairment but favorable retinal structural outcomes from the Early Treatment for Retinopathy of Prematurity (ETROP) study. J AAPOS.

[49] Frondas-Chauty A, Simon L, Branger B, Gascoin G, Flamant C, Ancel P (2014). Early growth and neurodevelopmental outcome in very preterm infants: impact of gender. Arch Dis Child Fetal Neonatal Ed.

[50] Burnett AC, Cheong JLY, Doyle LW (2018). Biological and social influences on the neurodevelopmental outcomes of preterm infants. Clin Perinatol.

