# Supplementary material for: Sex differences in the risk of retinopathy of prematurity: a systematic review, frequentist and Bayesian meta-analysis, and meta-regression
Source: World J Pediatr. 2023 Nov 27;20(4):340–52. doi: 10.1007/s12519-023-00775-x (PMC11052874; doi:10.1007/s12519-023-00775-x)
Supplement: Supplementary file 1 — (PDF 1500 KB) [file 12519_2023_775_MOESM1_ESM.pdf]

Supplementary materials

Sex differences in risk of retinopathy of prematurity: a systematic review, frequentist and Bayesian meta-analysis, and meta-regression

Supplementary Table 1. Search strategy

**PubMed**<br>((Sex[MESH] OR Sex Characteristics[MESH] OR Sex Distribution[MESH] OR "Sex Characteristic*"[tiab] OR "Gender Difference*"[tiab] OR "Sex Dimorphism*"[tiab] OR "Gender Characteristic*"[tiab] OR "Sex Difference*"[tiab] OR "Sex Distribution*"[tiab] OR "Gender Distribution*"[tiab] OR "Male Disadvantage"[tiab] OR "Female Advantage"[tiab] OR risk [tiab]) AND (Retinopathy of prematurity[MESH] OR "ROP"[tiab] OR " Retrolental fibroplasia "[tiab]))
**Embase**<br>((exp Sex Characteristics/ OR exp Sex Distribution / OR exp Sex Factors/) OR ("male disadvantage" or "female advantage" or gender or sex or "gender difference" or "gender differences" or "sex difference" or "sex differences" or "gender differential" or "gender differentials" or "sex differential" or "sex differentials" or "sexual dimorphism" or "sexually dimorphic")) AND (retinopathy of prematurity))

Supplementary Table 2. Characteristics of the included studies and risk of bias assessment

Author	Median year cohort	Country or region	Study design	Prospective	Total, *N*	Mean GA cohort	GA ≤ 28 weeks?	Subgroup(s) ROP	%ROP total cohort	NOS			
										Selection	Comparability	Outcome	Total
Abrishami et al., 2013 [1]	2008	Iran	Cohort	No	124	30.5	No	Any ROP	27.4	4	1	3	8
Adio et al., 2014 [2]	2012	Nigeria	Cohort	Yes	53	29.4	No	Any ROP	47.2	4	1	3	8
Adriono et al., 2006 [3]	2004	Indonesia	Cohort	Yes	33	31.6	No	Any ROP<br>Severe ROP	30.3<br>9.1	4	2	3	9
Aggarwal et al., 2002 [4]	2000	India	Cohort	Yes	76	30.8	No	Any ROP	31.6	3	1	3	7
Ahmadpour-Kacho et al., 2014 [5]	2009	Iran	Cohort	No	155	30.3	No	Any ROP	45.2	4	2	3	9
Ahmed et al., 2008 [6]	2001	Bangladesh	Cohort	Yes	114	31.3	No	Any ROP<br>Severe ROP	4.4<br>1.8	4	2	3	9
Ahmedhussain et al., 2021 [7]	2017	Saudi Arabia	Cohort	No	119	28.2	No	Any ROP	21.8	4	1	3	8
Akdogan et al., 2018 [8]	2017	Turkey	Cohort	No	458	30.1	No	Any ROP	46.9	4	1	3	8
Akkawi et al., 2019 [9]	2016	Palestine	Cohort	No	115	30.4	No	Any ROP	23.5	4	1	3	8
Akter et al., 2010 [10]	2007	Bangladesh	Cohort	Yes	58	29.6	Yes	Any ROP	39.7	4	2	3	9
Akyüz-Ünsal et al., 2019 [11]	2014	Turkey	Cohort	No	320	31.5	No	Any ROP	30.9	4	2	3	9
Al-Essa et al., 2000 [12]	1997	Kuwait	Cohort	Yes	234	30.2	No	Any ROP<br>Severe ROP	64.5<br>14.5	4	1	3	8
Ali et al., 2013 [13]	2011	Brunei	Cohort	No	67	29.5	No	Any ROP	34.3	4	1	2	7

Author	Median year cohort	Country or region	Study design	Prospective	Total, *N*	Mean GA cohort	GA ≤ 28 weeks?	Subgroup(s) ROP	%ROP total cohort	NOS			
										Selection	Comparability	Outcome	Total
Ali et al., 2017 [14]	2015	Egypt	Cohort	No	108	31.7	No	Any ROP<br>Severe ROP	69.4<br>10.2	4	2	3	9
Ali et al., 2019 [15]	2018	Pakistan	Cohort	No	60	32.7	No	Any ROP	33.3	4	2	3	9
Alizadeh et al., 2015 [16]	2008	Iran	Cohort	No	310	32.0	No	Any ROP	20.6	4	2	3	9
Alpay et al., 2012 [17]	2007	Turkey	Cohort	No	330	31.0	No	Any ROP<br>Severe ROP	37.6<br>6.1	4	1	3	8
Amer et al., 2012 [18]	2010	Saudi Arabia	Cohort	No	386	30.0	No	Any ROP<br>Severe ROP	23.3<br>6.5	4	1	2	7
Amrawanshi et al., 2019 [19]	2018	India	Cohort	Yes	178	31.4	No	Any ROP	33.7	4	1	3	8
Andújar Coba et al., 2009 [20]	2000	Cuba	Cohort	No	220	34.6	No	Any ROP	31.8	4	2	3	9
Anuk-İnce et al., 2013 [21]	2010	Turkey	Cohort	Yes	126	28.7	No	Severe ROP	27.0	4	1	3	8
Araz-Ersan et al., 2013 [22]	2003	Turkey	Cohort	No	2950	31.2	No	Severe ROP	15.8	4	2	3	9
Aydemir et al., 2011 [23]	2010	Turkey	Cohort	Yes	300	29.2	No	Severe ROP	7.0	4	1	3	8
Babaei et al., 2012 [24]	2010	Iran	Cohort	Yes	84	31.9	No	Any ROP	13.1	4	2	3	9
Bancalari et al., 2000 [25]	1991	Chile	Cohort	Yes	248	30.7	No	Any ROP	28.2	4	1	3	8
Barzilay et al., 2019 [26]	2006	Israel	Cohort	No	135	N	No	Any ROP	16.3	4	2	3	9
Bas et al., 2018 [27]	2017	Turkey	Cohort	Yes	2417	29.3	No	Severe ROP	16.3	4	2	3	9

Author	Median year cohort	Country or region	Study design	Prospective	Total, *N*	Mean GA cohort	GA ≤ 28 weeks?	Subgroup(s) ROP	%ROP total cohort	NOS			
										Selection	Comparability	Outcome	Total
Binenbaum et al., 2011 [28]	2002	USA/Canada	Cohort	Yes	367	26.3	Yes	Severe ROP	18.3	4	1	3	8
Binenbaum et al., 2012 [29]	2007	USA	Cohort	No	504	27.9	No	Severe ROP	4.0	4	1	3	8
Binet et al., 2012 [30]	2011	Canada	Cohort	No	2744	25.7	Yes	Severe ROP	22.0	4	2	3	9
Boghossian et al., 2018 [31]	2011	USA	Cohort	No	154872	26.0	No	Severe ROP	8.9	4	2	3	9
Borroni et al., 2013 [32]	2009	Italy	Cohort	No	401	26	Yes	Any ROP	61.6	4	2	3	9
Braimah et al., 2020 [33]	2019	Ghana	Cohort	Yes	401	32.3	No	Any ROP	13.7	4	2	3	9
Brown et al., 1998 [34]	1993	USA	Cohort	No	157	29.5	No	Any ROP	45.9	4	1	3	8
Carranza et al., 2019 [35]	2017	Peru	Cohort	No	216	32.4	No	Any ROP	33.3	4	2	3	9
Celebi et al., 2014 [36]	2012	Turkey	Cohort	No	235	27.1	No	Any ROP<br>Severe ROP	73.6<br>36.6	4	1	3	8
Chen et al., 2011 [37]	2002	USA	Cohort	No	622	26.6	No	Any ROP	47.1	4	2	3	9
Chen (ELGAN) et al., 2011 [38]	2003	USA	Cohort	No	1069	26	Yes	Any ROP<br>Severe ROP	72.8<br>15.0	4	1	3	8
Chen et al., 2015 [39]	2011	China	Cohort	Yes	468	32.3	No	Any ROP	12.4	4	2	3	9
Chiang et al., 2004 [40]	1998	USA	Cohort	No	15691	N	No	Any ROP	14.6	4	2	3	9
Choi et al., 2013 [41]	2008	Korea	Cohort	No	314	29.3	No	Any ROP<br>Severe ROP	27.4<br>12.7	4	1	3	8

Author	Median year cohort	Country or region	Study design	Prospective	Total, *N*	Mean GA cohort	GA ≤ 28 weeks?	Subgroup(s) ROP	%ROP total cohort	NOS			
										Selection	Comparability	Outcome	Total
Choi et al., 2014 [42]	2011	Korea	Cohort	No	185	31.1	No	Any ROP	12.4	4	1	3	8
Choo et al., 2009 [43]	2004	Malaysia	Cohort	Yes	70	27.4	Yes	Severe ROP	32.9	4	1	3	8
Çömez et al., 2021 [44]	2017	Turkey	Cohort	Yes	159	30.0	No	Any ROP<br>Severe ROP	48.4	4	1	3	8
Coskun et al., 2017 [45]	2014	Turkey	Cohort	Yes	127	30.2	No	Any ROP	33.9	4	1	3	8
Coutinho et al., 2017 [46]	2010	Portugal	Cohort	No	527	29	No	Any ROP	31.3	3	1	3	7
Dame et al., 2018 [47]	2003	Germany	Cohort	No	213	24.7	Yes	Severe ROP	33.8	4	2	3	9
Dammann et al., 2009 [48]	2003	Germany	Cohort	No	73	28.5	No	Any ROP<br>Severe ROP	60.3<br>17.8	4	2	3	9
Dani et al., 2001 [49]	1997	Italy	Cohort	Yes	45	28.8	No	Any ROP	46.7	4	2	3	9
Darlow et al., 2005 [50]	1999	Australia/New Zeeland	Cohort	Yes	2105	27.0	Yes	Severe ROP	9.6	4	2	3	9
Deepak et al., 2021 [51]	2019	India	Cohort	Yes	203	31.0	No	Any ROP	20.2	4	1	3	8
Di Fiore et al., 2010 [52]	2007	USA	Cohort	No	79	26.2	Yes	Severe ROP	20.3	4	1	3	8
Doifode et al., 2020 [53]	2018	India	Cohort	Yes	96	34.0	No	Any ROP	13.5	4	1	3	8
Doig Turkowsky et al., 2007 [54]	2003	Peru	Cohort	No	136	30.5	No	Any ROP<br>Severe ROP	70.6<br>19.1	4	2	3	9
Dutta et al., 2004 [55]	2000	India	Cohort	No	108	30.1	No	Severe ROP	50.9	4	2	3	9

Author	Median year cohort	Country or region	Study design	Prospective	Total, *N*	Mean GA cohort	GA ≤ 28 weeks?	Subgroup(s) ROP	%ROP total cohort	NOS			
										Selection	Comparability	Outcome	Total
Ebrahim et al., 2010 [56]	2006	Iran	Cohort	No	173	32.2	No	Any ROP	19.1	4	2	3	9
Englert et al., 2001 [57]	1995	USA	Cohort	No	87	25.5	Yes	Severe ROP	26.4	4	1	3	8
Enomoto et al., 2015 [58]	2012	Japan	Cohort	No	143	29.4	No	Severe ROP	16.8	4	2	3	9
Fajolu et al., 2015 [59]	2013	Nigeria	Cohort	Yes	80	28.9	No	Any ROP	15.0	4	1	3	8
Feghhi et al., 2012 [60]	2008	Iran	Cohort	No	576	31.6	No	Any ROP	31.8	4	2	3	9
Figueras-Aloy et al., 2010 [61]	2005	Spain	Cohort	No	718	29.7	No	Any ROP<br>Severe ROP	31.3<br>5.0	4	1	3	8
Flores-Nava et al., 2009 [62]	2004	Mexico	Cohort	No	207	29.0	No	Any ROP	54.1	3	1	3	7
Fortes Filho et al., 2011 [63]	2006	Brazil	Cohort	Yes	324	29.7	No	Any ROP<br>Severe ROP	29.9<br>7.4	4	2	3	9
Freitas et al., 2018 [64]	2011	Brazil	Cohort	No	602	30.7	No	Any ROP<br>Severe ROP	33.9<br>5.0	4	2	3	9
Gagliardi et al., 2021 [65]	2011	Multi	Cohort	No	20917	26.9	Yes	Severe ROP	7.8	4	2	3	9
García et al., 2018 [66]	2011	Mexico	Cohort	No	326	29.0	No	Any ROP	47.9	4	1	3	8
Gebeşçe et al., 2016 [67]	2009	Turkey	Cohort	No	219	32.8	No	Any ROP	21.9	4	2	3	9
Giapros et al., 2011 [68]	2004	Greece	Cohort	No	189	29.9	Yes	Any ROP	12.7	4	2	3	9
Giraldo et al., 2011 [69]	2006	Colombia	Cohort	No	1080	31.5	No	Any ROP	17.4	4	1	3	8

Author	Median year cohort	Country or region	Study design	Prospective	Total, *N*	Mean GA cohort	GA ≤ 28 weeks?	Subgroup(s) ROP	%ROP total cohort	NOS			
										Selection	Comparability	Outcome	Total
Goldstein et al., 2019 [70]	2009	USA	Cohort	No	14440	28.9	No	Severe ROP	12.9	4	1	3	8
Gonçalves et al., 2014 [71]	2010	Brazil	Cohort	Yes	110	29.2	No	Any ROP	44.5	4	2	3	9
Gu et al., 2011 [72]	2007	China	Cohort	No	553	32.5	No	Any ROP	9.8	4	1	3	8
Gupta et al., 2004 [73]	2001	India	Cohort	Yes	60	32.5	No	Any ROP	21.7	4	2	3	9
Hakeem et al., 2012 [74]	2010	Egypt	Cohort	Yes	172	33	No	Any ROP	19.2	3	1	3	7
Hanuna et al., 2021 [75]	2013	Slovenia	Cohort	No	205	26.0	Yes	Any ROP<br>Severe ROP	23.4<br>6.8	4	1	3	8
Hartnett et al., 2014 [76]	2006	USA	Cohort	No	1397	26.6	No	Any ROP<br>Severe ROP	57.4<br>10.0	4	2	3	9
Hellgren et al., 2021 [77]	2014	Sweden	Cohort	Yes	78	25.2	Yes	Any ROP<br>Severe ROP	78.2<br>39.7	4	1	3	8
Hernández et al., 2008 [78]	2004	Spain	Cohort	Yes	115	29.7	No	Severe ROP	15.7	4	1	3	8
Hintz et al., 2006 [79]	1999	USA	Cohort	Yes	2466	25.4	Yes	Severe ROP	15.5	4	2	3	9
Hirabayashi et al., 2010 [80]	2006	Japan	Cohort	No	86	28.9	No	Severe ROP	31.4	4	2	3	9
Holmström et al., 1996 [81]	1999	Sweden	Cohort	No	202	29	No	Any ROP	40.1	3	1	3	7
Hsieh et al., 2012 [82]	2006	Taiwan Province of China	Cohort	Yes	77	29.8	No	Any ROP<br>Severe ROP	50.6<br>23.4	4	1	3	8

Author	Median year cohort	Country or region	Study design	Prospective	Total, *N*	Mean GA cohort	GA ≤ 28 weeks?	Subgroup(s) ROP	%ROP total cohort	NOS			
										Selection	Comparability	Outcome	Total
Huang et al., 2012 [83]	2002	Taiwan Province of China	Cohort	No	195	27.9	No	Severe ROP	41.0	4	1	3	8
Huang et al., 2015 [84]	2002	Taiwan Province of China	Cohort	No	5718	29.8	No	Any ROP<br>Severe ROP	36.5<br>12.2	4	2	3	9
Huang et al., 2020 [85]	2017	China	Cohort	No	107	29.7	No	Any ROP	21.5	4	1	3	8
Hudalla et al., 2020 [86]	2007	Germany	Cohort	No	722	27.4	No	Any ROP	52.8	4	2	3	9
Hwang et al., 2015 [87]	2013	Korea	Cohort	Yes	2009	26.5	No	Any ROP<br>Severe ROP	34.1<br>11.6	4	1	3	8
Ikeda et al., 2004 [88]	1998	Japan	Cohort	No	45	28	No	Severe ROP	60.0	4	2	3	9
Ingolfsland et al., 2020 [89]	2014	USA	Cohort	Yes	83	28.3	No	Any ROP	24.1	4	1	3	8
Isaza et al., 2013 [90]	2008	Canada	Cohort	No	383	27.4	No	Any ROP	44.6	4	2	3	9
Ito et al., 2017 [91]	2008	Japan	Cohort	No	38023	28.0	No	Severe ROP	14.5	4	2	3	9
Kabataş et al., 2013 [92]	2012	Turkey	Cohort	Yes	113	30.5	No	Any ROP<br>Severe ROP	46.9<br>15.9	4	1	3	8
Kaempf et al., 2011 [93]	2005	USA	Cohort	No	382	28.0	No	Any ROP<br>Severe ROP	34.6<br>7.6	4	2	3	9
Kalmeh et al., 2013 [94]	2012	Iran	Cohort	Yes	111	26.9	No	Any ROP<br>Severe ROP	40.5<br>13.5	4	1	3	8

Author	Median year cohort	Country or region	Study design	Prospective	Total, *N*	Mean GA cohort	GA ≤ 28 weeks?	Subgroup(s) ROP	%ROP total cohort	NOS			
										Selection	Comparability	Outcome	Total
Kang et al., 2018 [95]	2006	Taiwan Province of China	Cohort	No	11180	N	No	Any ROP	36.6	3	2	3	8
Karkhaneh et al., 2008 [96]	2005	Iran	Cohort	No	953	31.1	No	Any ROP	31.1	2	2	3	7
Karlowicz et al., 2000 [97]	1995	USA	Cohort	No	449	25.9	No	Severe ROP	12.9	4	1	3	8
Karna et al., 2005 [98]	1997	USA	Cohort	No	576	30.0	No	Any ROP<br>Severe ROP	47.7<br>7.8	4	1	3	8
Kavurt et al., 2014 [99]	2012	Turkey	Cohort	Yes	495	29.3	No	Severe ROP	5.9	4	1	3	8
Kaya et al., 2013 [100]	2008	Turkey	Cohort	No	123	29.6	No	Any ROP<br>Severe ROP	74.8<br>34.1	4	1	3	8
Kent et al., 2012 [101]	2001	Australia	Cohort	No	2002	26.3	yes	Severe ROP	12.0	4	2	3	9
Khorshidifar et al., 2019 [102]	2017	Iran	Cohort	Yes	207	32.6	No	Any ROP<br>Severe ROP	33.3<br>11.1	4	2	3	9
Kim et al., 2012 [103]	2007	Korea	Cohort	No	140	28.0	No	Severe ROP	47.1	4	1	3	8
Kim et al., 2015 [104]	2008	Korea	Cohort	No	211	28.0	No	Any ROP<br>Severe ROP	42.2<br>22.7	4	2	3	9
Kim et al., 2018 [105]	2015	Korea	Cohort	No	402	28.5	No	Any ROP<br>Severe ROP	24.1<br>11.4	4	2	3	9
Knežević et al., 2011 [106]	2007	Serbia	Cohort	No	317	32.3	No	Any ROP	39.1	4	1	3	8

Author	Median year cohort	Country or region	Study design	Prospective	Total, *N*	Mean GA cohort	GA ≤ 28 weeks?	Subgroup(s) ROP	%ROP total cohort	NOS			
										Selection	Comparability	Outcome	Total
						32.7		Severe ROP	21.5				
Kong et al., 2012 [107]	2008	Korea	Cohort	No	121	24.0	Yes	Severe ROP	64.5	4	1	3	8
Kossambe et al., 2019 [108]	2017	India	Cohort	Yes	244	32.1	No	Any ROP<br>Severe ROP	15.2<br>5.7	4	1	3	8
Kumar et al., 2011 [109]	2006	India	Cohort	No	704	31.0	No	Severe ROP	4.7	4	2	3	9
Kurtul et al., 2015 [110]	2013	Turkey	Cohort	No	100	28.1	No	Any ROP	80.0	4	1	3	8
Lad et al., 2009 [111]	2001	USA	Cohort	No	435683	N	No	Any ROP	13.5	4	1	3	8
Le et al., 2016 [112]	2011	India	Cohort	No	66	31.0	No	Severe ROP	12.1	4	1	3	8
Lee (ELGAN) et al., 2013 [113]	2003	USA	Cohort	Yes	1199	25.6	Yes	Severe ROP	13.5	4	1	3	8
Leng et al., 2018 [114]	2015	China	Cohort	No	436	33.8	No	Any ROP<br>Severe ROP	31.7<br>14.0	4	2	3	9
Li et al., 2013 [115]	2005	Taiwan Province of China	Cohort	No	503	29.1	No	Any ROP	37.8	4	1	3	8
Liu et al., 2014 [116]	2011	China	Cohort	No	459	31.7	No	Any ROP	20.0	4	1	3	8
Liu et al., 2012 [117]	2004	Taiwan Province of China	Cohort	No	96	26.8	No	Any ROP<br>Severe ROP	92.7<br>74.0	4	1	3	8
Lorenz et al., 2009 [118]	2004	Germany	Cohort	Yes	1222	30.0	No	Any ROP	27.6	4	1	3	8
Lundgren et al., 2018 [119]	2015	Australia	Cohort	No	227	26.4	No	Severe ROP	11.0	4	2	3	9

Author	Median year cohort	Country or region	Study design	Prospective	Total, *N*	Mean GA cohort	GA ≤ 28 weeks?	Subgroup(s) ROP	%ROP total cohort	NOS			
										Selection	Comparability	Outcome	Total
Lundgren et al., 2019 [120]	2014	Sweden	Cohort	Yes	87	25.5	Yes	Severe ROP	25.3	4	1	3	8
Lundgren et al., 2014 [121]	2006	Sweden & USA	Cohort	No	2941	27.7	No	Severe ROP	10.0	4	2	3	9
Månsson (EXPRESS) et al., 2015 [122]	2006	Sweden	Cohort	No	398	31.4	No	Severe ROP	33.9	4	2	3	9
Martínez-Cruz et al., 2012 [123]	2004	Mexico	Cohort	Yes	139	29	No	Any ROP	24.5	4	1	3	8
Mayock (PENUT) et al., 2020 [124]	2015	USA	Cohort	Yes	845	25.5	Yes	Any ROP<br>Severe ROP	59.5<br>9.1	4	1	3	8
Mishra et al., 2019 [125]	2017	India	Cohort	Yes	360	29.8	No	Any ROP	11.7	4	2	3	9
Mitsiakos et al., 2016 [126]	2001	USA	Cohort	No	1389	28.9	No	Any ROP	15.6	4	1	3	8
Mohamed et al., 2013 [127]	2005	USA	Cohort	No	582		No	Any ROP	29.2	4	1	3	8
Mutangana et al., 2020 [128]	2016	Rwanda	Cohort	Yes	417	31.7	No	Any ROP<br>Severe ROP	7.4<br>3.1	3	2	3	8
Mutlu et al., 2008 [129]	2002	Turkey	Cohort	No	318	31	No	Any ROP	37.1	4	1	3	8
Neubauer et al., 2012 [130]	2006	Austria	Cohort	No	408	28	No	Severe ROP	3.9	4	1	3	8
Ni et al., 2020 [131]	2014	China	Cohort	No	184	29.7	No	Severe ROP	18.5	4	1	3	8
Nødgaard et al., 1996 [132]	1992	Denmark	Cohort	No	139	29.8	No	Any ROP	18.7	4	2	3	9
Noyola et al., 2002 [133]	1994	USA	Cohort	No	92	25.8	Yes	Severe ROP	38.0	4	1	3	8
Owen et al., 2017 [134]	2013	USA	Cohort	No	457		No	Any ROP	52.5	4	2	3	9

Author	Median year cohort	Country or region	Study design	Prospective	Total, *N*	Mean GA cohort	GA ≤ 28 weeks?	Subgroup(s) ROP	%ROP total cohort	NOS			
										Selection	Comparability	Outcome	Total
						28.1		Severe ROP	11.6				
Ozdemir et al., 2012 [135]	2010	Turkey	Cohort	Yes	206	27.9	No	Severe ROP	12.1	4	1	3	8
Ozturk et al., 2021 [136]	2015	Turkey	Cohort	No	120	28.9	No	Any ROP<br>Severe ROP	71.7<br>28.3	4	1	3	8
Paranjpe et al., 2019 [137]	2018	India	Cohort	No	233	32	No	Any ROP	35.6	4	1	3	8
Parupia et al., 2001 [138]	1998	USA	Cohort	No	130	26.5	Yes	Severe ROP	52.3	4	1	3	8
Peacock et al., 2012 [139]	2000	UK	Cohort	No	797	26.5	Yes	Severe ROP	10.7	4	2	3	9
Pérez-Muñuzuri et al., 2010 [140]	2007	Spain	Cohort	Yes	74	30.8	No	Any ROP	32.4	4	1	3	8
Pinheiro et al., 2009 [141]	2005	Brasil	Cohort	No	663	31.9	No	Any ROP	50.4	4	2	3	9
Pivodic et al., 2020 [142]	2013	Sweden/USA/Germany	Cohort	No	7609	28.1	No	Any ROP<br>Severe ROP	31.9<br>5.8	4	1	3	8
Poovichayasumlit et al., 2020 [143]	2017	Thailand	Cohort	Yes	100	31.0	No	Any ROP	10.0	4	1	3	8
Porcelli et al., 2010 [144]	2003	USA	Cohort	Yes	77	26.3	Yes	Severe ROP	14.3	4	1	3	8
Port et al., 2014 [145]	2006	USA	Cohort	No	1354	28.7	No	Any ROP	38.8	4	1	3	8
Quinn (CRYO-ROP) et al., 2016 [146]	1987	USA	Cohort	Yes	4099	27.9	No	Any ROP<br>Severe ROP	63.6<br>17.8	4	1	3	8
Quinn (e-ROP) et al., 2016 [146]	2012	USA	Cohort	Yes	1257	27	No	Any ROP	63.7	4	1	3	8

Author	Median year cohort	Country or region	Study design	Prospective	Total, *N*	Mean GA cohort	GA ≤ 28 weeks?	Subgroup(s) ROP	%ROP total cohort	NOS			
										Selection	Comparability	Outcome	Total
								Severe ROP	19.3				
Quinn (ET-ROP) et al., 2016 [146]	2001	USA	Cohort	Yes	6998	27.4	No	Any ROP<br>Severe ROP	68.0<br>14.3	4	1	3	8
Rasoulinejad et al., 2016 [147]	2010	Iran	Cohort	No	680	31.5	No	Any ROP	45.0	4	2	3	9
Rasyidah et al., 2013 [148]	2011	Indonesia	Cohort	No	48	30.6	No	Any ROP	12.5	4	1	3	8
Rivera-Rueda et al., 2020 [149]	2017	Mexico	Cohort	Yes	282	28.8	No	Any ROP	53.9	4	1	3	8
Ruiz Cano, 1999 [150]	1995	Spain	Cohort	No	193	32.4	No	Any ROP	15.5	3	1	3	7
Sabzehei et al., 2013 [151]	2009	Iran	Cohort	No	414	30.5	No	Any ROP	13.8	4	2	3	9
Saeidi et al., 2009 [152]	2006	Iran	Cohort	Yes	47	29.0	No	Any ROP	8.5	4	1	2	7
Santana Hernández et al., 2018 [153]	2011	Spain	Cohort	No	683	29	No	Any ROP	29.0	4	1	3	8
Sarikabadayi et al., 2011 [154]	2009	Turkey	Cohort	Yes	371	30.0	No	Any ROP<br>Severe ROP	53.6<br>5.9	4	1	3	8
Sathar et al., 2018 [155]	2014	India	Cohort	Yes	203	30.9	No	Severe ROP	27.6	4	1	3	8
Serenius et al., 2004 [156]	1995	Sweden	Cohort	No	140	24.5	Yes	Severe ROP	81.0	4	2	3	9
Shah et al., 2005 [157]	2000	Singapore	Cohort	No	408	29.7	No	Severe ROP	6.9	4	1	3	8
Shim et al., 2017 [158]	2014	Korea	Cohort	No	1854	27.2	No	Severe ROP	17.7	4	2	3	9
Shinwell et al., 2007 [159]	1999	Israel	Cohort	No	8858	28.6	No	Severe ROP	7.2	4	2	3	9

Author	Median year cohort	Country or region	Study design	Prospective	Total, *N*	Mean GA cohort	GA ≤ 28 weeks?	Subgroup(s) ROP	%ROP total cohort	NOS			
										Selection	Comparability	Outcome	Total
Silveira et al., 2011 [160]	2006	Brazil	Cohort	Yes	74	29.6	Yes	Any ROP<br>Severe ROP	33.8	4	2	3	9
Singh et al., 2016 [161]	2011	India	Cohort	Yes	64	31.2	No	Any ROP	21.9	4	1	3	8
Skiöld et al., 2014 [162]	2006	Sweden	Cohort	No	107	25.5	Yes	Severe ROP	18.7	4	1	3	8
Slidsborg et al., 2016 [163]	2004	Denmark	Cohort	No	6490	30	No	Severe ROP	2.5	4	1	3	8
Sood et al., 2012 [164]	2010	India	Cohort	No	158	32.3	No	Any ROP	45.6	3	2	3	8
Sood et al., 2010 [165]	2005	USA	Cohort	Yes	877	26	Yes	Any ROP<br>Severe ROP	69.6<br>25.2	4	1	3	8
Reyes et al., 2017 [166]	2009	Oman	Cohort	No	171	29.2	No	Any ROP	40.4	3	1	3	7
Stanković-Babić et al., 2014 [167]	2007	Serbia	Cohort	No	93	34.7	No	Any ROP	22.6	4	1	3	8
Sveinsdóttir et al., 2018 [168]	2006	Sweden	Cohort	Yes	52	26.5	No	Any ROP	36.5	4	1	3	8
Sun et al., 2021 [169]	2014	China	Cohort	No	3587	28.7	No	Any ROP<br>Severe ROP	11.6<br>5.6	4	1	3	8
Tadesse et al., 2002 [170]	1996	USA	Cohort	No	161	26.4	Yes	Any ROP	70.8	4	1	3	8
Taqui et al., 2008 [171]	2005	Pakistan	Cohort	No	68	29.8	No	Any ROP	32.4	3	1	3	7
Teoh et al., 1995 [172]	1991	Malaysia	Cohort	Yes	113	31.6	No	Any ROP	31.9	3	1	3	7
Thomas et al., 2015 [173]	2007	Canada	Cohort	No	9187	27.6	No	Any ROP	12.7	4	2	3	9

Author	Median year cohort	Country or region	Study design	Prospective	Total, *N*	Mean GA cohort	GA ≤ 28 weeks?	Subgroup(s) ROP	%ROP total cohort	NOS			
										Selection	Comparability	Outcome	Total
Tioseco et al., 2006 [174]	1998	USA	Cohort	No	833	30.9	No	Any ROP	28.2	4	2	3	9
Todd et al., 1990 [175]	1987	Australia	Cohort	Yes	16	27	No	Severe ROP	50.0	4	0	2	6
Todd et al., 2012 [176]	2000	Australia	Cohort	No	303	25.5	Yes	Any ROP<br>Severe ROP	67.3<br>22.4	4	1	3	8
Tolia et al., 2020 [177]	2014	USA	Cohort	Yes	323	24.7	Yes	Severe ROP	34.7	4	1	3	8
Tsui et al., 2013 [178]	2008	USA	Cohort	No	450	28.7	No	Severe ROP	12.9	4	1	3	8
Uchida et al., 2014 [179]	2008	Japan	Cohort	Yes	182	29.1	No	Any ROP<br>Severe ROP	46.2<br>24.7	4	2	3	9
Ueda et al., 2020 [180]	2014	Japan	Cohort	No	278	28.7<br>29.8	No	Any ROP<br>Severe ROP	43.2<br>6.5	4	1	3	8
Ugurbas et al., 2010 [181]	2007	Turkey	Cohort	No	260	30.3<br>30.5	No	Any ROP<br>Severe ROP	23.1<br>11.5	4	1	3	8
Van der Merwe et al., 2013 [182]	2010	South Africa	Cohort	No	356	28.4	No	Severe ROP	4.2	3	2	3	8
Van Sorge et al., 2014 [183]	2009	The Netherlands	Cohort	No	1379	29.8	No	Any ROP	21.2	4	2	3	9
Vasavada et al., 2017 [184]	2013	India	Cohort	Yes	280	30.6	No	Any ROP<br>Severe ROP	19.3<br>10.0	4	2	3	9
Vucinovic et al., 2018 [185]	2008	Croatia	Cohort	No	247	31.0	No	Any ROP	23.9	4	1	3	8

Author	Median year cohort	Country or region	Study design	Prospective	Total, *N*	Mean GA cohort	GA ≤ 28 weeks?	Subgroup(s) ROP	%ROP total cohort	NOS			
										Selection	Comparability	Outcome	Total
								Severe ROP	9.3				
Wang et al., 2015 [186]	2012	China	Cohort	Yes	303	29.5	No	Any ROP	26.4	4	2	3	9
Wang et al., 2018 [187]	2011	Taiwan Province of China	Cohort	No	4941	28.5	No	Severe ROP	15.8	4	1	3	8
Wang (SUNDROP) et al., 2015 [188]	2008	USA	Cohort	Yes	608	28.8	Yes	Severe ROP	3.6	4	1	3	8
Wani et al., 2013 [189]	2007	Kuwait	Cohort	No	207	28.1	No	Severe ROP	24.2	4	1	3	8
Wikstrand et al., 2011 [190]	2007	Sweden	Cohort	No	46	25.7	Yes	Severe ROP	50.0	4	1	3	8
Woo et al., 2012 [191]	2007	Korea	Cohort	No	246	29.2	No	Any ROP<br>Severe ROP	33.3<br>11.0	4	2	3	9
Wu et al., 2018 [192]	2014	China	Cohort	No	504	30.6	No	Any ROP<br>Severe ROP	26.0<br>3.2	4	2	3	9
Yang et al., 2011 [193]	2006	Taiwan Province of China	Cohort	No	216	29.1	No	Any ROP<br>Severe ROP	45.8<br>19.0	3	2	3	8
Yang et al., 2017 [194]	2014	China	Cohort	No	10	29.2	No	Any ROP	60.0	4	2	3	9
Yang et al., 2007 [195]	2001	USA	Cohort	No	503	26.7	Yes	Any ROP<br>Severe ROP	59.4<br>7.0	4	2	3	9
Yanovitch et al., 2006 [196]	2003	USA	Cohort	No	259	30.5	No	Any ROP	4.2	4	1	3	8
Yau et al., 2015 [197]	2010	China	Cohort	No	513	30.0	No	Any ROP	18.5	4	2	3	9

Author	Median year cohort	Country or region	Study design	Prospective	Total, *N*	Mean GA cohort	GA ≤ 28 weeks?	Subgroup(s) ROP	%ROP total cohort	NOS			
										Selection	Comparability	Outcome	Total
								Severe ROP					
Ying (G-ROP) et al., 2019 [198]	2009	USA	Cohort	No	7483	28.0	No	Any ROP	43.1	4	2	3	9
Yu et al., 2012 [199]	2006	USA	Cohort	No	8758	30	No	Any ROP	11.7	4	1	3	8
Yu (G-ROP) et al., 2021 [200]	2005	USA	Cohort	No	11463	28.4	No	Severe ROP	5.9	4	2	3	9
Zarei et al., 2019 [201]	2017	Iran	Cohort	No	1990	32.3	No	Any ROP	28.9	4	2	3	9
Zepeda et al., 2014 [202]	2012	Mexico	Cohort	No	59	29.7	No	Severe ROP	28.8	4	2	3	9
Zisk et al., 2011 [203]	2005	USA	Cohort	No	12807	29.4	No	Severe ROP	2.2	4	1	3	8

ROP retinopathy of prematurity, *GA* gestational age, *NOS* Newcastle-Ottawa scale

Supplementary Table 3. Data on heterogeneity of the Bayesian model-average meta-analysis of the association between infant sex and any retinopathy of prematurity

Variables	Groups	K	Heterogeneity (tau)	Standard dev.	Credible interval		BF_{rf}	Evidence for		Frequentist *P* value for heterogeneity
					Lower limit	Upper limit		Random effects	Fixed effects	
**GA**	All	144	0.127	0.016	0.099	0.160	$> 10^{12}$	Extreme		< 0.001
	≤ 28 wk	12	0.111	0.042	0.049	0.210	2.183	Weak		0.018
	> 28 wk	132	0.132	0.017	0.102	0.167	$> 10^{12}$	Extreme		< 0.001
**(sub)Continent**	Africa	6	0.204	0.150	0.048	0.605	0.835		Weak	0.111
	Eastern Asia	24	0.089	0.033	0.038	0.165	0.534		Weak	0.247
	Central-Southeast Asia	33	0.178	0.061	0.079	0.313	72.0	Very strong		0.003
	Western Asia	20	0.116	0.033	0.062	0.191	548.2	Extreme		0.018
	Europe	20	0.092	0.039	0.038	0.187	0.518		Weak	0.237
	North America	25	0.127	0.026	0.083	0.186	$> 10^{12}$	Extreme		< 0.001
	Latin America	14	0.197	0.055	0.110	0.326	$> 10^{5}$	Extreme		< 0.001
**SDI**	High	52	0.110	0.019	0.077	0.151	$> 10^{12}$	Extreme		< 0.001
	High-middle	31	0.105	0.028	0.058	0.170	266.4	Extreme		0.046
	Middle	40	0.186	0.037	0.123	0.268	$> 10^{6}$	Extreme		< 0.001
	Middle-low/low	20	0.155	0.081	0.046	0.352	0.974		Weak	0.189

GA gestational age, *SDI* sociodemographic index, *BF* Bayes factor

Supplementary Table 4. Data on heterogeneity of the Bayesian model-average meta-analysis of the association between infant sex and severe retinopathy of prematurity

Variables	Groups	K	Heterogeneity (tau)	Standard dev.	Credible interval		BF_{rf}	Evidence for		*P* value frequentist analysis
					Lower limit	Upper limit		Random effects	Fixed effects	
**GA**	All	113	0.130	0.020	0.097	0.174	$> 10^{12}$	Extreme		< 0.001
	≤ 28 wk	28	0.085	0.034	0.036	0.169	0.188	Extreme		< 0.001
	> 28 wk	85	0.148	0.025	0.106	0.203	$> 10^{12}$	Very strong		0.006
**(sub)Continent**	Africa	3	0.239	0.233	0.048	0.851	0.859		Weak	0.550
	Eastern Asia	25	0.207	0.046	0.135	0.310	$> 10^{12}$		Weak	0.305
	Central-Southeast Asia	12	0.124	0.070	0.041	0.305	0.350		Moderate	0.653
	Western Asia	18	0.135	0.068	0.045	0.301	0.978	Moderate		0.026
	Europe	16	0.117	0.057	0.041	0.256	0.473	Strong		0.002
	North America	27	0.070	0.025	0.033	0.129	0.409	Extreme		< 0.001
	Latin America	5	0.176	0.124	0.045	0.506	0.652		Weak	0.927
	Oceania	4	0.194	0.146	0.049	0.594	1.028	Strong		0.005
**SDI**	High	69	0.134	0.022	0.097	0.183	$> 10^{12}$		Moderate	0.140
	High-middle	21	0.118	0.054	0.043	0.253	0.658		Moderate	0.672
	Middle	15	0.142	0.076	0.044	0.332	0.609		Moderate	0.638
	Middle-low/low	8	0.164	0.110	0.045	0.460	0.572	Moderate		0.016

GA gestational age, *SDI* sociodemographic index, *BF* Bayes factor

Supplementary Table 5. Data on heterogeneity of the Bayesian model-average meta-analysis of the association between infant sex and non-severe retinopathy of prematurity

Variables	Groups	K	Heterogeneity (tau)	Standard dev.	Credible interval		BF_{rf}	Evidence for		*P* value frequentist analysis
					Lower limit	Upper limit		Random effects	Fixed effects	
**GA**	All	52	0.157	0.034	0.098	0.232	688,892	Extreme		< 0.001
	≤ 28 wk	7	0.223	0.114	0.072	0.513	7.125	Moderate		0.003
	> 28 wk	45	0.152	0.036	0.089	0.229	126,974	Extreme		< 0.001
**(sub)Continent**	Africa	2	0.204	0.178	0.047	0.689	0.777		Weak	0.459
	Eastern Asia	13	0.143	0.066	0.049	0.296	0.856		Weak	0.148
	Central-Southeast Asia	6	0.220	0.158	0.048	0.645	1.105	Weak		0.328
	Western Asia	10	0.128	0.063	0.043	0.279	0.678		Weak	0.350
	Europe	6	0.185	0.145	0.046	0.504	0.920		Weak	0.142
	North America	10	0.198	0.062	0.105	0.345	45277	Extreme		< 0.001
	Latin America	3	0.236	0.183	0.051	0.712	1.105	Weak		0.077
**SDI**	High	26	0.174	0.043	0.102	0.270	300,479	Extreme		< 0.001
	High-middle	12	0.099	0.047	0.038	0.220	0.280		Moderate	0.853
	Middle	10	0.227	0.105	0.071	0.473	5.856	Moderate		0.010
	Middle-low/low	4	0.198	0.156	0.046	0.611	0.776		Weak	0.843

GA gestational age, *SDI* sociodemographic index, *BF* Bayes factor

Supplementary Table 6. Meta-regression of the correlation between different covariates and the risk ratio of the association of infant sex and ROP

**Outcomes**	**Covariate**	**K**	**Point estimate**	**Lower limit**	**Upper limit**	***P* value**	**R^2-analog**
Any ROP	Median year of cohort (all studies)	144	0.000	-0,005	0,005	0.920	0.0
	Median year of cohort (time span of study ≤ 5 y)	123	-0.002	-0.007	0.003	0.377	0.0
	GA total cohort	140	0.000	-0.008	0.009	0.916	0.0
Severe ROP	Median year of cohort (all studies)	113	-0.004	-0.014	0.006	0.385	0.0
	Median year of cohort (time span of study ≤ 5 y)	91	-0.003	-0.015	0.008	0.557	0.0
	GA total cohort	113	-0.011	-0.043	0.020	0.418	0.0
Non-severe ROP	Median year of cohort (all studies)	52	0.000	-0.008	0.008	0.943	0.0
	Median year of cohort (time span of study ≤ 5 y)	38	0.002	-0.007	0.011	0.947	0.0
	GA total cohort	52	0.015	-0.033	0.025	0.789	0.0

R^2 analog: total between-study variance explained by the moderator. *ROP* retinopathy of prematurity, *GA* gestational age

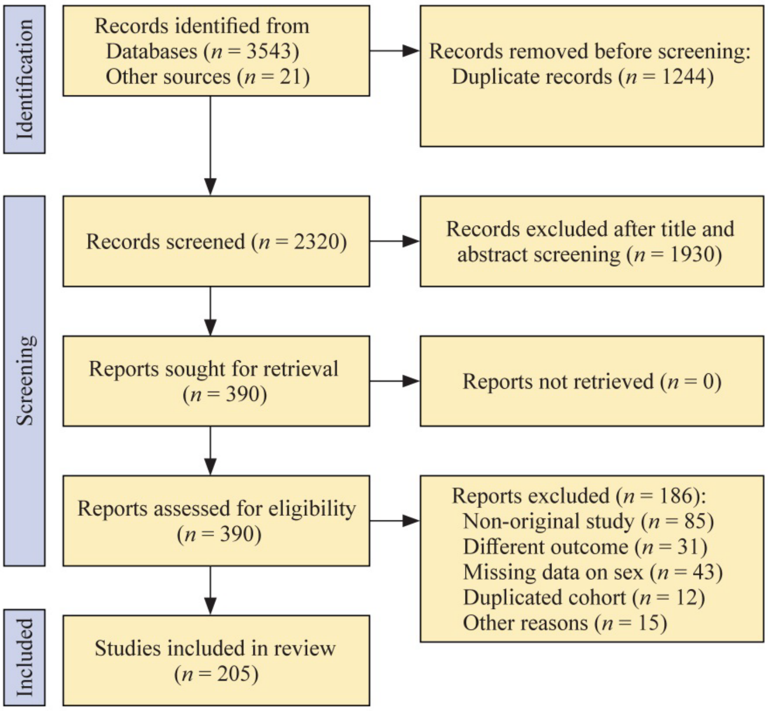

Supplementary Fig. 1 PRISMA flow diagram of the systematic search

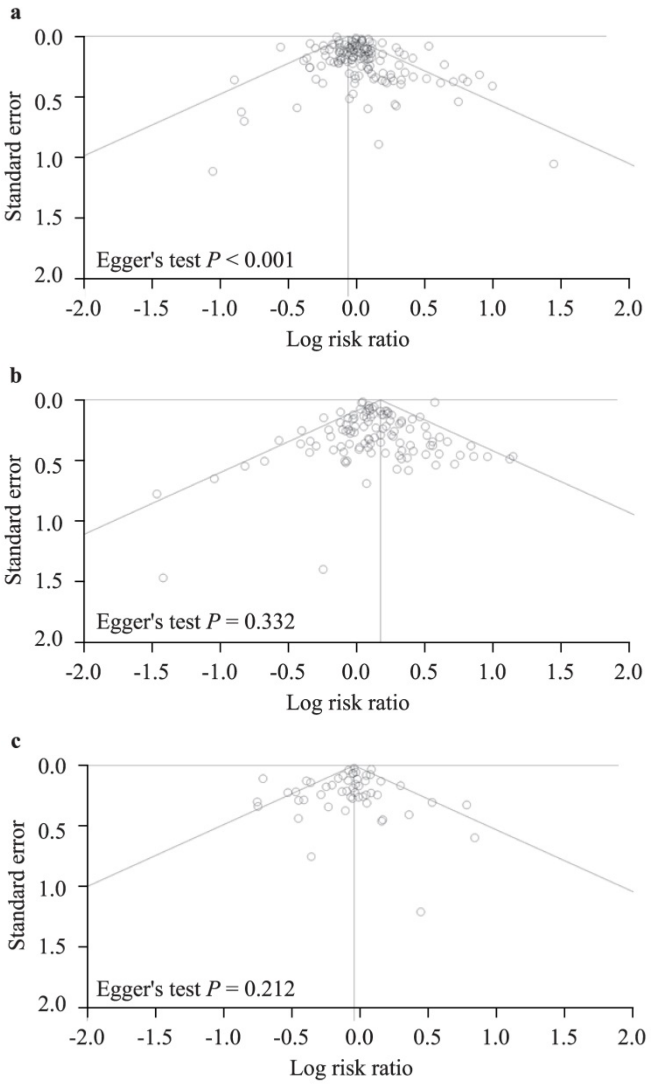

Supplementary Fig. 2 Funnel plot for publication bias analysis for the studies included in the different meta-analyses. **a** Any ROP; **b** severe ROP; **c** non-severe ROP. *ROP* retinopathy of prematurity

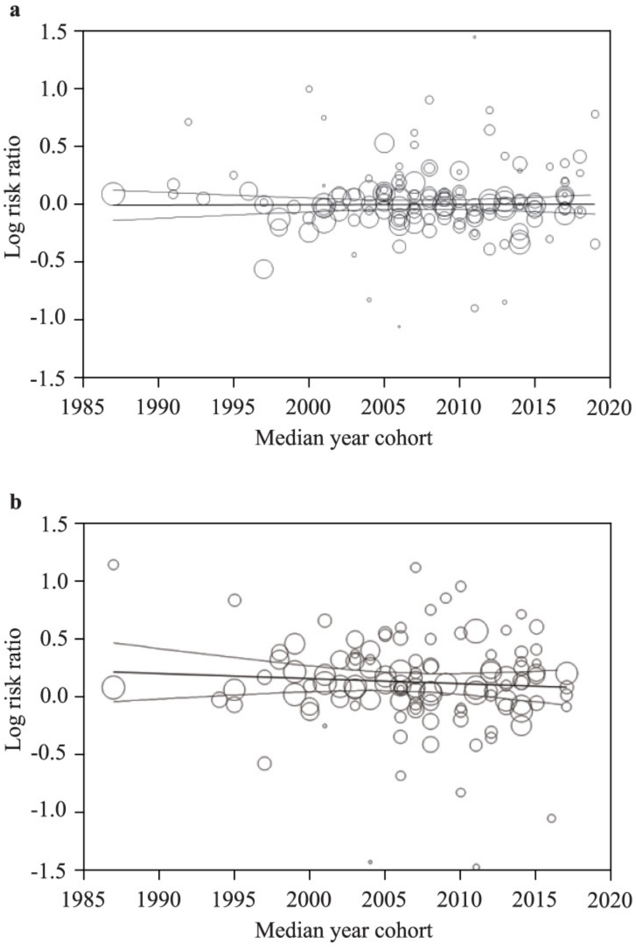

Supplementary Fig. 3 Meta regression. Plot showing the correlation between the association of male sex with any ROP (**a**) and severe ROP (**b**) and the median year of birth of the infants of each cohort. The size of the circles represents the relative weight of each study. *ROP* retinopathy of prematurity

References

1. Abrishami M, Maemori GA, Boskabadi H, Yaeghobi Z, Mafi-Nejad S, Abrishami M. Incidence and risk factors of retinopathy of prematurity in mashhad, northeast iran. Iran Red Crescent Med J. 2013;15:229-33.
2. Adio AO, Ugwu RO, Nwokocha CG, Eneh AU. Retinopathy of prematurity in port harcourt, Nigeria. ISRN Ophthalmol. 2014;2014:481527.
3. Adriono GA, Sitorus R. Screening for retinopathy of prematurity at Cipto Mangunkusumo Hospital, Jakarta, Indonesia–a preliminary report. Acta Med Litu. 2006.
4. Aggarwal R, Deorari AK, Azad R, Kumar H, Talwar D, Sethi A, et al. Changing profile of retinopathy of prematurity. Journal of tropical pediatrics. 2002;48:239-42.
5. Ahmadpour-Kacho M, Jashni Motlagh A, Rasoulinejad SA, Jahangir T, Bijani A, Zahed Pasha Y. Correlation between hyperglycemia and retinopathy of prematurity. Pediatr Int. 2014;56:726-30.
6. Ahmed ANU, Muslima H, Anwar KS, Khan NZ, Chowdhury MA, Saha SK, et al. Retinopathy of prematurity in Bangladeshi neonates. J Trop Pediatr. 2008;54:333-9.
7. Ahmedhussain HK, Khayyat WW, Aldhahwani BM, Aljuwaybiri AO, Badeeb NO, Khan MA, et al. Retinopathy of prematurity: incidence and perinatal risk factors in a tertiary hospital in Saudi Arabia. J Clin Neonatol. 2021;10:31.
8. Akdogan M, Sabaner MC, Karabas VL. Epidemiological and clinical features of the preterms followed-up at our clinic in Turkey: a study about 458 infants. J Ophthalmol. 2018;8:214-23.
9. Akkawi MT, Shehadeh MM, Shams ANA, Al-Hardan DM, Omar LJ, Almahmoud OH, et al. Incidence and risk factors of retinopathy of prematurity in three neonatal intensive care units in Palestine. BMC Ophthalmol. 2019;19:189.
10. Akter S, Hossain MM, Shirin M, Khalil I, Anwar KS. Blood transfusion: a risk factor in retinopathy of prematurity. Bangladesh J Child Health. 2010;34:38-43.
11. Akyüz-Ünsal Aİ, Key Ö, Güler D, Bekmez S, Sagus M, Akcan AB, et al. Retinopathy of prematurity risk factors: does human milk prevent retinopathy of prematurity? Turk J Pediatr. 2019;61:13-9.
12. Al-Essa M, Azad R, Rashwan N. Threshold stage of retinopathy of prematurity: maternal and neonatal risk factors. Ann Saudi Med. 2000;20:129-31.
13. Ali NAM, George J, Joshi N, Chong E. Prevalence of retinopathy of prematurity in Brunei Darussalam. Int J Ophthalmol. 2013;6:381-4.
14. Ali AA, Gomaa NA, Awadein AR, Al-Hayouti HH, Hegazy AI. Retrospective cohort study shows that the risks for retinopathy of prematurity included birth age and weight, medical conditions and treatment. Acta Paediatr. 2017;106:1919-27.
15. Association with risk facors of retinopathy of prematurity (ROP) of in hospital newborns of low birth weight (LBW) in tertiary care hospital, Ali MA, Anwar M, Naeem MM. Retinopathy of prematurity (ROP). Profession Med J. 2019. https://doi.org/10.29309/TPMJ/2019.26.04.3375.
16. Alizadeh Y, Zarkesh M, Moghadam RS, Esfandiarpour B, Behboudi H, Karambin MM, et al. Incidence and risk factors for retinopathy of prematurity in North of Iran. J Ophthalmic Vis Res. 2015;10:424-8.
17. Alpay A, Ugurbas SH. Incidence and risk factors for retinopathy of prematurity in the West Black Sea region, Turkey. Turk J Pediatr. 2012;54:113-8.
18. Amer M, Jafri WH, Nizami AM, Shomrani AI, Al-Dabaan AA, Rashid K. Retinopathy of prematurity: are we missing any infant with retinopathy of prematurity? Br J Ophthalmol. 2012;96:1052-5.
19. Amrawanshi S, Patel S. An analysis of incidence and risk factors for retinopathy of prematurity in a tertiary care center of central India. IOSR-JDMS. 2019;18:37-41.

20. Andújar Coba P, Mier Armas M, Coba MJ, Pérez Torga JE. Susceptible factors of retinopathy in premature infants in Praia city. Cuban J Ophthalmol. 2009;22:97-110 (**in Spanish**).
21. Anuk-İnce D, Gülcan H, Hanta D, Ecevit Ae, Akkoyun İ, Kurt A, et al. Poor postnatal weight gain predicts stage 3+ retinopathy of prematurity in very low birth weight infants. Turk J Pediatr. 2013;55:304-8.
22. Araz-Ersan B, Kir N, Akarcay K, Aydinoglu-Candan O, Sahinoglu-Keskek N, Demirel A, et al. Epidemiological analysis of retinopathy of prematurity in a referral centre in Turkey. Br J Ophthalmol. 2013;97:15-7.
23. Aydemir O, Sarikabadayi Y, Aydemir C, Tunay Z, Tok L, Erdeve O, et al. Adjusted poor weight gain for birth weight and gestational age as a predictor of severe ROP in VLBW infants. Eye (Lond). 2011;25:725-9.
24. Babaei H, Ansari MR, Alipour AA, Ahmadipour S, Safari-Faramani R, Vakili J. Incidence and risk factors for retinopathy of prematurity in very low birth weight infants in Kermanshah, Iran. World Appl Sci J. 2012;18:600-4.
25. Bancalari A, González R, Vásquez C. Retinopathy of prematurity: incidence rate and related factors. Chilean J Pediatr. 2000;71:114-21 (**in Spanish**).
26. Barzilay B, Shirman N, Bibi H, Abu-Kishk I. Newborn gender as a predictor of neonatal outcome in mixed gender twins born with very low birth weight. BMC Pediatr. 2019;19:328.
27. Bas AY, Demirel N, Koc E, Isik DU, Hirfanoglu İM, Tunc T. Incidence, risk factors and severity of retinopathy of prematurity in Turkey (TR-ROP study): a prospective, multicentre study in 69 neonatal intensive care units. Br J Ophthalmol. 2018;102:1711-6.
28. Binenbaum G, Ying G, Quinn GE, Dreiseitl S, Karp K, Roberts RS, et al. A clinical prediction model to stratify retinopathy of prematurity risk using postnatal weight gain. Pediatrics. 2011;127:e607-14.
29. Binenbaum G, Ying G, Quinn GE, Huang J, Dreiseitl S, Antigua J, et al. The CHOP postnatal weight gain, birth weight, and gestational age retinopathy of prematurity risk model. Arch Ophthalmol. 2012;130:1560-5.
30. Binet ME, Bujold E, Lefebvre F, Tremblay Y, Piedboeuf B, Network CN. Role of gender in morbidity and mortality of extremely premature neonates. Am J Perinatol. 2012;29:159-66.
31. Boghossian NS, Geraci M, Edwards EM, Horbar JD. Sex differences in mortality and morbidity of infants born at less than 30 weeks' gestation. Pediatrics. 2018;142:e20182352.
32. Borroni C, Carlevaro C, Morzenti S, De Ponti E, Bozzetti V, Console V, et al. Survey on retinopathy of prematurity (ROP) in Italy. Ital J Pediatr. 2013;39:43.
33. Braimah IZ, Enweronu-Laryea C, Sackey AH, Kenu E, Agyabeng K, Ofori-Adjei ID, et al. Incidence and risk factors of retinopathy of prematurity in Korle-Bu Teaching Hospital: a baseline prospective study. BMJ Open. 2020;10:e035341.
34. Brown BA, Thach AB, Song JC, Marx JL, Kwun RC, Frambach DA. Retinopathy of prematurity: evaluation of risk factors. Int Ophthalmol. 1998;22:279-83.
35. Carranza Mendizábal CS, Díaz Manrique M. Neonatal risk factors associated with retinopathy in preterm infants at the Ounanu National Hipolito Hospital from January 2016 to December 2018. 2019 (**in Spanish**).
36. Celebi ARC, Petricli IS, Hekimoglu E, Demirel N, Bas AY. The incidence and risk factors of severe retinopathy of prematurity in extremely low birth weight infants in Turkey. Med Sci Monit. 2014;20:1647-53.
37. Chen M, Çitil A, McCabe F, Leicht KM, Fiascone J, Dammann CE, et al. Infection, oxygen, and immaturity: interacting risk factors for retinopathy of prematurity. Neonatology. 2011;99:125-32.
38. Chen ML, Allred EN, Hecht JL, Onderdonk A, VanderVeen D, Wallace DK, et al. Placenta microbiology and histology and the risk for severe retinopathy of prematurity. Invest Ophthalmol Vis Sci. 2011;52:7052-8.

39. Chen Y, Xun D, Wang YC, Wang B, Geng SH, Chen H, et al. Incidence and risk factors of retinopathy of prematurity in two neonatal intensive care units in North and South China. Chin Med J (Engl). 2015;128:914-8.
40. Chiang MF, Arons RR, Flynn JT, Starren JB. Incidence of retinopathy of prematurity from 1996 to 2000: analysis of a comprehensive New York state patient database. Ophthalmology. 2004;111:1317-25.
41. Choi JH, Löfqvist C, Hellström A, Heo H. Efficacy of the screening algorithm WINROP in a Korean population of preterm infants. JAMA Ophthalmol. 2013;131:62-6.
42. Choi JY, Han YI, Kim JH, Kim ES, Jeon J. The most important factors for retinopathy of prematurity in preterm infants. Korean J Perinatol. 2014;25:153-8.
43. Choo MM, Martin FJ, Theam LC, U-Teng C. Retinopathy of prematurity in extremely low birth weight infants in Malaysia. J AAPOS. 2009;13:446-9.
44. Çömez A, Yurttutan S, Akkececi NS, Bozkaya A, Kökülsarı G, Evgin İ, et al. Red cell distribution width and its association with retinopathy of prematurity. Int Ophthalmol. 2021;41:699-706.
45. Coşkun Y, Dalkan C, Yabaş Ö, Demirel ÖÜ, Bayar ES, Sakarya S, et al. A predictive score for retinopathy of prematurity by using clinical risk factors and serum insulin-like growth factor-1 levels. Int J Ophthalmol. 2017;10:1722-7.
46. Coutinho I, Pedrosa C, Mota M, Azeredo-Lopes S, Santos C, Pires G, et al. Retinopathy of prematurity: results from 10 years in a single neonatal intensive care unit. JPNIM. 2017;6:e060122.
47. Dame C, Sciesielski LK, Rau C, Badur CA, Bührer C. The erythropoietin promoter variant rs1617640 is not associated with severe retinopathy of prematurity, independent of treatment with erythropoietin. J Pediatr. 2018;199:256-9.
48. Dammann O, Brinkhaus MJ, Bartels DB, Dördelmann M, Dressler F, Kerk J, et al. Immaturity, perinatal inflammation, and retinopathy of prematurity: a multi-hit hypothesis. Early Hum Dev. 2009;85:325-9.
49. Dani C, Reali M, Bertini G, Martelli E, Pezzati M, Rubaltelli F. The role of blood transfusions and iron intake on retinopathy of prematurity. Early Hum Dev. 2001;62:57-63.
50. Darlow BA, Hutchinson JL, Henderson-Smart DJ, Donoghue DA, Simpson JM, Evans NJ. Prenatal risk factors for severe retinopathy of prematurity among very preterm infants of the Australian and New Zealand Neonatal Network. Pediatrics. 2005;115:990-6.
51. Deepak M, Poonam S, Mehta J, Aparna G. Incidence and risk factors predisposing to retinopathy of prematurity and treatment outcome: a retrospective cohort study. 2021. https://doi.org/10.13140/RG.2.2.20525.20965.
52. Di Fiore JM, Bloom JN, Orge F, Schutt A, Schluchter M, Cheruvu VK, et al. A higher incidence of intermittent hypoxemic episodes is associated with severe retinopathy of prematurity. J Pediatr. 2010;157:69-73.
53. Doifode SV, Ashtekar SD, Mirgunde SP, Khot S. An assessment of associated risk factors for retinopathy of prematurity (ROP) among neonates of NICU of a tertiary care hospital: a cohort study. 2020.
54. Doig Turkowsky J, Cervantes AC, Rocha SPV, Torres SRV, Vega SRV, Balladares SPV, et al. Incidence rate and evolution of retinopathy of prematurity in very low birth weight surviving children graduated from Lima Institute of Perinatal Maternity and Infant. Rev Peru Pediatr. 2007;60:88-92 (**in Spanish**).
55. Dutta S, Narang S, Narang A, Dogra M, Gupta A. Risk factors of threshold retinopathy of prematurity. Indian Pediatr. 2004;41:665-72.
56. Ebrahim M, Ahmad RS, Mohammad M. Incidence and risk factors of retinopathy of prematurity in Babol, North of Iran. Ophthalmic Epidemiol. 2010;17:166-70.
57. Englert JA, Saunders RA, Purohit D, Hulsey TC, Ebeling M. The effect of anemia on retinopathy of prematurity in extremely low birth weight infants. J Perinatol. 2001;21:21-6.

58. Enomoto H, Miki A, Matsumiya W, Honda S. Evaluation of oxygen supplementation status as a risk factor associated with the development of severe retinopathy of prematurity. Ophthalmologica. 2015;234:135-8.
59. Fajolu I, Rotimi-Samuel A, Aribaba O, Musa K, Akinsola F, Ezeaka V, et al. Retinopathy of prematurity and associated factors in Lagos, Nigeria. Paediatr Int Child Health. 2015;35:324-8.
60. Feghhi M, Altayeb SMH, Haghi F, Kasiri A, Farahi F, Dehdashtyan M, et al. Incidence of retinopathy of prematurity and risk factors in the south-western region of Iran. Middle East Afr J Ophthalmol. 2012;19:101-6.
61. Figueras-Aloy J, Álvarez-Domínguez E, Morales-Ballus M, Salvia-Roiges M, Moretones-Suñol G. Early administration of erythropoietin in the extreme premature, a risk factor for retinopathy of prematurity? An Pediatr (Barc). 2010;73:327-33 (**in Spanish**).
62. Flores-Nava G, Barrera-Vázquez CN, de la Fuente-Torres MA, Torres-Narváez P. Retinopathy of premature infants. Identify some risk factors. Mexican Children's Hospital Medical Bulletin. 2009;66:425-30.
63. Fortes Filho JB, Costa MC, Eckert GU, Santos PG, Silveira RC, Procianoy RS. Maternal preeclampsia protects preterm infants against severe retinopathy of prematurity. J Pediatr. 2011;158:372-6.
64. Freitas AM, Mörschbächer R, Thorell MR, Rhoden EL. Incidence and risk factors for retinopathy of prematurity: a retrospective cohort study. Int J Retina Vitreous. 2018;4:20.
65. Gagliardi L, Rusconi F, Reichman B, Adams M, Modi N, Lehtonen L, et al. Neonatal outcomes of extremely preterm twins by sex pairing: an international cohort study. Arch Dis Child Fetal Neonatal Ed. 2021;106:17-24.
66. García H, González-Cabello H, Soriano-Beltrán CA, Soto-Dávila MA, Vázquez-Lara Y, Hernández-Galván C. Frequency and severity of retinopathy of prematurity in a neonatal intensive care unit. Gac Med Mex. 2018;154:475-81.
67. Gebeşçe A, Uslu H, Keleş E, Yildirim A, Gürler B, Yazgan H, et al. Retinopathy of prematurity: incidence, risk factors, and evaluation of screening criteria. Turk J Med Sci. 2016;46:315-20.
68. Giapros V, Drougia A, Asproudis I, Theocharis P, Andronikou S. Low gestational age and chronic lung disease are synergistic risk factors for retinopathy of prematurity. Early Hum Dev. 2011;87:653-7.
69. Giraldo Restrepo MM, Hurtado Guzmán A, Donado Gómez JH, Molina Betancur MC. Epidemiology of retinopathy in Medellin premature infants, 2003-2008. Iatreia. 2011:250-8.
70. Goldstein GP, Leonard SA, Kan P, Koo EB, Lee HC, Carmichael SL. Prenatal and postnatal inflammation-related risk factors for retinopathy of prematurity. J Perinatol. 2019;39:964-73.
71. Gonçalves E, Násser LS, Martelli DR, Alkmim IR, Mourão TV, Caldeira AP, et al. Incidence and risk factors for retinopathy of prematurity in a Brazilian reference service. Sao Paulo Med J. 2014;132:85-91.
72. Gu MH, Jin J, Yuan TM, Yu HM. Risk factors and outcomes for retinopathy of prematurity in neonatal infants with a birth weight of 1,501–2,000 g in a Chinese Neonatal Unit. Med Princ Pract. 2011;20:244-7.
73. Gupta VP, Dhaliwal U, Sharma R, Gupta P, Rohatgi J. Retinopathy of prematurity—risk factors. Indian J Pediatr. 2004;71:887-92.
74. Hakeem AH, Mohamed GB, Othman MF. Retinopathy of prematurity: a study of prevalence and risk factors. Middle East Afr J Ophthalmol. 2012;19(3):289-94.
75. Hanuna S, Rus M, Štucin Gantar I, Erčulj V, Tekavčič Pompe M, Grosek Š. Noninvasive ventilation for respiratory distress syndrome is a potential risk factor for retinopathy of prematurity: Single Slovenian tertiary center study. Wien Klin Wochenschr. 2021;133:687-94.

76. Hartnett ME, Morrison MA, Smith S, Yanovitch TL, Young TL, Colaizy T, et al. Genetic variants associated with severe retinopathy of prematurity in extremely low birth weight infants. Invest Ophthalmol Vis Sci. 2014;55:6194-203.
77. Hellgren G, Lundgren P, Pivodic A, Löfqvist C, Nilsson AK, Ley D, et al. Decreased platelet counts and serum levels of VEGF-A, PDGF-BB, and BDNF in extremely preterm infants developing severe ROP. Neonatology. 2021;118:18-27.
78. Hernández M, Orduña C, Bosch V, Salinas R, Alcaraz JL, Marín JM. Retinopathy of prematurity in the Murcia region of Spain. Incidence and severity. Arch Soc Esp Oftalmol. 2008;83:423-8 (**in Spanish**).
79. Hintz SR, Kendrick DE, Vohr BR, Poole WK, Higgins RD, Nichd Neonatal Research Network. Gender differences in neurodevelopmental outcomes among extremely preterm, extremely-low-birthweight infants. Acta Paediatr. 2006;95:1239-48.
80. Hirabayashi H, Honda S, Morioka I, Yokoyama N, Sugiyama D, Nishimura K, et al. Inhibitory effects of maternal smoking on the development of severe retinopathy of prematurity. Eye (Lond). 2010;24(6):1024-7.
81. Holmström G, Thomassen P, Broberger U. Maternal risk factors for retinopathy of prematurity–a population-based study. Acta Obstet Gynecol Scand. 1996;75:628-35.
82. Hsieh CJ, Liu JW, Huang JS, Lin KC. Refractive outcome of premature infants with or without retinopathy of prematurity at 2 years of age: a prospective controlled cohort study. Kaohsiung J Med Sci. 2012;28:204-11.
83. Huang HM, Lin SA, Chang YC, Kuo HK. Correlation between periventricular leukomalacia and retinopathy of prematurity. Eur J Ophthalmol. 2012;22:980-4.
84. Huang HC, Yang HI, Chou HC, Chen CY, Hsieh WS, Tsou KI, et al. Preeclampsia and retinopathy of prematurity in very-low-birth-weight infants: a population-based study. PLoS One. 2015;10:e0143248.
85. Huang HB, Chen YH, Wu J, Hicks M, Yi YZ, Zhang QS, et al. Early risk factors for retinopathy of prematurity in very and extremely preterm Chinese neonates. Front Pediatr. 2020;8:553519.
86. Hudalla H, Bruckner T, Pöschl J, Strowitzki T, Kuon RJ. Antenatal exposure to fenoterol is not associated with the development of retinopathy of prematurity in infants born before 32 weeks of gestation. Arch Gynecol Obstet. 2020;301:687-92.
87. Hwang JH, Lee EH, Kim EAR. Retinopathy of prematurity among very-low-birth-weight infants in Korea: incidence, treatment, and risk factors. J Korean Med Sci. 2015;30(Suppl 1):S88-94.
88. Ikeda H, Kuriyama S. Risk factors for retinopathy of prematurity requiring photocoagulation. Jpn J Ophthalmol. 2004;48:68-71.
89. Ingolfsland EC, Haapala JL, Buckley LA, Demarath EW, Guiang SF, Ramel SE. Late growth and changes in body composition influence odds of developing retinopathy of prematurity among preterm infants. Nutrients. 2020;12:78.
90. Isaza G, Arora S, Bal M, Chaudhary V. Incidence of retinopathy of prematurity and risk factors among premature infants at a neonatal intensive care unit in Canada. J Pediatr Ophthalmol Strabismus. 2013;50:27-32.
91. Ito M, Tamura M, Namba F, Japan NRNo. Role of sex in morbidity and mortality of very premature neonates. Pediatr Int. 2017;59:898-905.
92. Kabataş EU, Beken S, Aydın B, Dilli D, Zenciroğlu A, Okumuş N. The risk factors for retinopathy of prematurity and need of laser photocoagulation: a single center experience. Gazi Med J. 2013;24.
93. Kaempf JW, Kaempf AJ, Wu Y, Stawarz M, Niemeyer J, Grunkemeier G. Hyperglycemia, insulin and slower growth velocity may increase the risk of retinopathy of prematurity. J Perinatol. 2011;31:251-7.

94. Kalmeh ZA, Azarpira N, Mosallaei M, Hosseini H, Malekpour Z. Genetic polymorphisms of vascular endothelial growth factor and risk for retinopathy of prematurity in South of Iran. Mol Biol Rep. 2013;40:4613-8.
95. Kang EYC, Lien R, Wang NK, Lai CC, Chen KJ, Hwang YS, et al. Retinopathy of prematurity trends in Taiwan of China: a 10-year nationwide population study. Invest Ophthalmol Vis Sci. 2018;59:3599-607.
96. Karkhaneh R, Mousavi SZ, Riazi-Esfahani M, Ebrahimzadeh SA, Roohipoor R, Kadivar M, et al. Incidence and risk factors of retinopathy of prematurity in a tertiary eye hospital in Tehran. Br J Ophthalmol. 2008;92:1446-9.
97. Karlowicz MG, Giannone PJ, Pestian J, Morrow AL, Shults J. Does candidemia predict threshold retinopathy of prematurity in extremely low birth weight (≤ 1000 g) neonates? Pediatrics. 2000;105:1036-40.
98. Karna P, Muttineni J, Angell L, Karmaus W. Retinopathy of prematurity and risk factors: a prospective cohort study. BMC Pediatr. 2005;5:18.
99. Kavurt S, Özcan B, Aydemir O, Bas AY, Demirel N. Risk of retinopathy of prematurity in small for gestational age premature infants. Indian Pediatr. 2014;51:804-6.
100. Kaya M, Çokakli M, Berk AT, Yaman A, Yesilirmak D, Kumral A, et al. Associations of VEGF/VEGF-receptor and HGF/c-Met promoter polymorphisms with progression/regression of retinopathy of prematurity. Curr Eye Res. 2013;38:137-42.
101. Kent AL, Wright IM, Abdel-Latif ME, New South Wales and Australian Capital Territory Neonatal Intensive Care Units Audit Group. Mortality and adverse neurologic outcomes are greater in preterm male infants. Pediatrics. 2012;129:124-31.
102. Khorshidifar M, Nikkhah H, Ramezani A, Entezari M, Daftarian N, Norouzi H, et al. Incidence and risk factors of retinopathy of prematurity and utility of the national screening criteria in a tertiary center in Iran. Int J Ophthalmol. 2019;12:1330-6.
103. KIM NH, LEE SM, EUN HS, PARK MS, PARK KI, Namgung R, et al. Risk of surgery for retinopathy of prematurity in very low birth weight infants. J Korean Soc Neonatol. 2012;19:71-6.
104. Kim J, Jin JY, Kim SS. Postnatal weight gain in the first two weeks as a predicting factor of severe retinopathy of prematurity requiring treatment. Korean J Pediatr. 2015;58:52-9.
105. Kim CY, Jung E, Kim EN, Kim CJ, Lee JY, Hwang JH, et al. Chronic placental inflammation as a risk factor of severe retinopathy of prematurity. J Pathol Transl Med. 2018;52:290-7.
106. Knežević S, Stojanović N, Oros A, Savić D, Simović A, Knežević J. Analysis of risk factors in the development of retinopathy of prematurity. Srp Arh Celok Lek. 2011;139:433-8.
107. Kong M, Shin DH, Kim SJ, Ham DI, Kang SW, Chang YS, et al. Retinopathy of prematurity in infants born before 25 weeks gestation in a Korean single neonatal intensive care unit: incidence, natural history and risk factors. J Korean Med Sci. 2012;27:1556-62.
108. Kossambe S, Joglekar S, D'Lima A, Silveira M. Incidence and risk factors of retinopathy of prematurity in Goa, India: a report from tertiary care centre. Int J Contemp Pediatr. 2019;6:1228-34.
109. Kumar P, Sankar MJ, Deorari A, Azad R, Chandra P, Agarwal R, et al. Risk factors for severe retinopathy of prematurity in preterm low birth weight neonates. Indian J Pediatr. 2011;78:812-6.
110. Kurtul BE, Kabatas EU, Zenciroglu A, Ozer PA, Ertugrul GT, Beken S, et al. Serum neutrophil-to-lymphocyte ratio in retinopathy of prematurity. J AAPOS. 2015;19:327-31.
111. Lad EM, Hernandez-Boussard T, Morton JM, Moshfeghi DM. Incidence of retinopathy of prematurity in the United States: 1997 through 2005. Am J Ophthalmol. 2009;148:451-8.
112. Le C, Basani LB, Zurakowski D, Ayyala RS, Agraharam SG. Retinopathy of prematurity: Incidence, prevalence, risk factors, and outcomes at a tertiary care center in Telangana. J Clin Ophthalmol Res. 2016;4:119.

113. Lee JW, McElrath T, Chen M, Wallace DK, Allred EN, Leviton A, et al. Pregnancy disorders appear to modify the risk for retinopathy of prematurity associated with neonatal hyperoxemia and bacteremia. J Matern Fetal Neonatal Med. 2013;26(8):811-8.
114. Leng Y, Huang W, Ren G, Cai C, Tan Q, Liang Y, et al. The treatment and risk factors of retinopathy of prematurity in neonatal intensive care units. BMC Ophthalmol. 2018;18:301.
115. Li ML, Hsu SM, Chang YS, Shih MH, Lin YC, Lin CH, et al. Retinopathy of prematurity in southern Taiwan of China: a 10-year tertiary medical center study. J Formos Med Assoc. 2013;112:445-53.
116. Liu Q, Yin ZQ, Ke N, Chen L, Chen XK, Fang J, et al. Incidence of retinopathy of prematurity in southwestern China and analysis of risk factors. Med Sci Monit. 2014;20:1442-51.
117. Liu YS, Chen TC, Yang CH, Yang CM, Huang JS, Ho TC, et al. Incidence, risk factors, and treatment of retinopathy of prematurity among very low birth body weight infants. Taiwan J Ophthalmol. 2012;2:60-3.
118. Lorenz B, Spasovska K, Elflein H, Schneider N. Wide-field digital imaging based telemedicine for screening for acute retinopathy of prematurity (ROP). Six-year results of a multicentre field study. Graefes Arch Clin Exp Ophthalmol. 2009;247:1251-62.
119. Lundgren P, Athikarisamy SE, Patole S, Lam GC, Smith LE, Simmer K. Duration of anaemia during the first week of life is an independent risk factor for retinopathy of prematurity. Acta Paediatr. 2018;107:759-66.
120. Lundgren P, Hellgren G, Pivodic A, Sävman K, Smith LE, Hellström A. Erythropoietin serum levels, versus anaemia as risk factors for severe retinopathy of prematurity. Pediatr Res. 2019;86:276-82.
121. Lundgren P, Kistner A, Andersson EM, Hansen Pupp I, Holmström G, Ley D, et al. Low birth weight is a risk factor for severe retinopathy of prematurity depending on gestational age. PLoS One. 2014;9:e109460.
122. Månsson J, Fellman V, Stjernqvist K, Group ES. Extremely preterm birth affects boys more and socio-economic and neonatal variables pose sex-specific risks. Acta Paediatr. 2015;104:514-21.
123. Martínez-Cruz CF, Salgado-Valladares M, Poblano A, Trinidad-Pérez MC. Risk factors associated with retinopathy of prematurity and visual alterations in infants with extremely low birth weight. Rev Invest Clin. 2012;64:136-43.
124. Mayock DE, Xie Z, Comstock BA, Heagerty PJ, Juul SE. High-dose erythropoietin in extremely low gestational age neonates does not alter risk of retinopathy of prematurity. Neonatology. 2020;117:650-7.
125. Mishra G, Modi M, Saluja S, Soni A, Khosla A. Incidence and risk factors of retinopathy of prematurity among very-low-birth-weight neonates. Curr Med Res Pract. 2019;9:215-7.
126. Mitsiakos G, Papageorgiou A. Incidence and factors predisposing to retinopathy of prematurity in inborn infants less than 32 weeks of gestation. Hippokratia. 2016;20:121-6.
127. Mohamed S, Murray JC, Dagle JM, Colaizy T. Hyperglycemia as a risk factor for the development of retinopathy of prematurity. BMC Pediatr. 2013;13:78.
128. Mutangana F, Muhizi C, Mudereva G, Noë P, Musiime S, Ngambe T, et al. Retinopathy of prematurity in Rwanda: a prospective multi-centre study following introduction of screening and treatment services. Eye (Lond). 2020;34:847-56.
129. Mutlu FM, Altinsoy HI, Mumcuoglu T, Kerimoglu H, Kilic S, Kul M, et al. Screening for retinopathy of prematurity in a tertiary care newborn unit in Turkey: frequency, outcomes, and risk factor analysis. J Pediatr Ophthalmol Strabismus. 2008;45:291-8.
130. Neubauer V, Griesmaier E, Ralser E, Kiechl-Kohlendorfer U. The effect of sex on outcome of preterm infants–a population-based survey. Acta Paediatr. 2012;101:906-11.
131. Ni YQ, Xu SS, Zhang T, Huang X. Clinical features and changes of disease spectrum of zone II retinopathy of prematurity: a 10-year review. Int J Ophthalmol. 2020;13(11):1753-7.

132. Nødgaard H, Andreasen H, Hansen H, Sørensen HT. Risk factors associated with retinopathy of prematurity (ROP) in northern Jutland, Denmark 1990-1993. Acta Ophthalmol Scand. 1996;74:306-10.
133. Noyola DE, Bohra L, Paysse EA, Fernandez M, Coats DK. Association of candidemia and retinopathy of prematurity in very low birthweight infants. Ophthalmology. 2002;109:80-4.
134. Owen LA, Morrison MA, Hoffman RO, Yoder BA, DeAngelis MM. Retinopathy of prematurity: a comprehensive risk analysis for prevention and prediction of disease. PLoS One. 2017;12:e0171467.
135. Ozdemır R, Sarı F, Tunay Z, Erdeve O, Canpolat F, Oguz S, et al. The association between respiratory tract Ureaplasma urealyticum colonization and severe retinopathy of prematurity in preterm infants≤ 1250 g. Eye (Lond). 2012;26:992-6.
136. Ozturk T, Durmaz Engin C, Kaya M, Yaman A. Complete blood count parameters to predict retinopathy of prematurity: when to evaluate and what do they tell us? Int Ophthalmol. 2021;41:2009-18.
137. Paranjpe G, Sarwate R, Shetty N. Risk factor and outcome of retinopathy of prematurity among premature babies admitted to tertiary care hospital: a retrospective observational study. Curr Med Res Opin. 2019;2:334-8.
138. Parupia MH, Dhanireddy R. Association of postnatal dexamethasone use and fungal sepsis in the development of severe retinopathy of prematurity and progression to laser therapy in extremely low-birth-weight infants. J Perinatol. 2001;21:242-7.
139. Peacock JL, Marston L, Marlow N, Calvert SA, Greenough A. Neonatal and infant outcome in boys and girls born very prematurely. Pediatr Res. 2012;71:305-10.
140. Pérez-Muñuzuri A, Fernández-Lorenzo J, Couce-Pico M, Blanco-Teijeiro M, Fraga-Bermúdez J. Serum levels of IGF1 are a useful predictor of retinopathy of prematurity. Acta Paediatr. 2010;99:519-25.
141. Pinheiro AM, da Silva WA, Bessa CGF, Cunha HM, Ferreira MÁF, Gomes AHB. Incidence and risk factors of retinopathy of prematurity at Onofre Lopes University Hospital, Natal (RN)-Brazil. Brazilian Arch Ophthalmol. 2009;72.
142. Pivodic A, Hård AL, Löfqvist C, Smith LE, Wu C, Bründer MC, et al. Individual risk prediction for sight-threatening retinopathy of prematurity using birth characteristics. JAMA Ophthalmol. 2020;138:21-9.
143. Poovichayasumlit C. Retinopathy of prematurity at Thammasat University Hospital. TMJ. 2020;20:297-306.
144. Porcelli PJ, Weaver RG Jr. The influence of early postnatal nutrition on retinopathy of prematurity in extremely low birth weight infants. Early Hum Dev. 2010;86:391-6.
145. Port AD, Chan RP, Ostmo S, Choi D, Chiang MF. Risk factors for retinopathy of prematurity: insights from outlier infants. Graefes Arch Clin Exp Ophthalmol. 2014;252:1669-77.
146. Quinn GE, Barr C, Bremer D, Fellows R, Gong A, Hoffman R, et al. Changes in course of retinopathy of prematurity from 1986 to 2013: comparison of three studies in the United States. Ophthalmology. 2016;123:1595-600.
147. Rasoulinejad SA, Montazeri M. Retinopathy of prematurity in neonates and its risk factors: a seven year study in Northern Iran. Open Ophthalmol J. 2016;10:17-21.
148. Rasyidah R, Fing SA. Incidence and risk factors of retinopathy of prematurity. Paediatr Indones. 2013;53:76-82.
149. Rivera-Rueda MA, Fernández-Carrocera LA, B Salgado-Valladares M, Cordero-González G, A Coronado-Zarco I, Cardona-Pérez JA. Retinopathy of prematurity, frequency and risk factors in very low birth weight infants. Bol Med Hosp Infant Mex. 2020;77:135-41.
150. Ruiz Cano R. Retinopathy in premature infants: epidemiological factors and prognosis. 1999.
151. Sabzehei MK, Afjeh SA, Dastjani Farahani A, Shamshiri AR, Esmaili F. Retinopathy of prematurity: incidence, risk factors, and outcome. Arch Iran Med. 2013;16:507-12.

152. Saeidi R, Hashemzadeh A, Ahmadi S, Rahmani S. Prevalence and predisposing factors of retinopathy of prematurity in very low-birth-weight infants discharged from NICU. Iranian J Pediatr. 2009;19:59-63.
153. Santana Hernández A, Cabrera Marrero B, Rodríguez Melián L, Reyes Súarez D, Castellano Solanes J, González Azpeitia G. Identify risk factors associated with retinopathy in premature infants. Descriptive research. Arch Canary Islands Ophthalmol Society. 2018.
154. Sarikabadayi YU, Aydemir O, Ozen ZT, Aydemir C, Tok L, Oguz SS, et al. Screening for retinopathy of prematurity in a large tertiary neonatal intensive care unit in Turkey: frequency and risk factors. Ophthalmic Epidemiol. 2011;18:269-74.
155. Sathar A, Shanavas A, Girijadevi P, Jasmin L, Kumar S, Pillai RK. Risk factors of retinopathy of prematurity in a tertiary care hospital in South India. Clin Epidemiol Glob Health. 2018;6:44-9.
156. Serenius F, Ewald U, Farooqi A, Holmgren PÅ, Håkansson S, Sedin G. Short-term outcome after active perinatal management at 23–25 weeks of gestation. A study from two Swedish perinatal centres. Part 3: neonatal morbidity. Acta Paediatr. 2004;93:1090-7.
157. Shah V, Yeo C, Ling Y, Ho L. Incidence, risk factors of retinopathy of prematurity among very low birth weight infants in Singapore. Ann Acad Med Singapore. 2005;34:169-78.
158. Shim SY, Cho SJ, Kong K, Park E. Gestational age-specific sex difference in mortality and morbidities of preterm infants: a nationwide study. Sci Rep. 2017;7:6161.
159. Shinwell ES, Reichman B, Lerner-Geva L, Boyko V, Blickstein I, Network IN. "Masculinizing" effect on respiratory morbidity in girls from unlike-sex preterm twins: a possible transchorionic paracrine effect. Pediatrics. 2007;120:e447-53.
160. Silveira RC, Fortes Filho JB, Procianoy RS. Assessment of the contribution of cytokine plasma levels to detect retinopathy of prematurity in very low birth weight infants. Invest Ophthalmol Vis Sci. 2011;52:1297-301.
161. Singh PH, Surana AU, Shah AN. Retinopathy of prematurity in neonatal care unit Int J Contemp Pediatr. 2016;3:234-9.
162. Skiöld B, Alexandrou G, Padilla N, Blennow M, Vollmer B, Ådén U. Sex differences in outcome and associations with neonatal brain morphology in extremely preterm children. J Pediatr. 2014;164:1012-8.
163. Slidsborg C, Jensen A, Forman JL, Rasmussen S, Bangsgaard R, Fledelius HC, et al. Neonatal risk factors for treatment-demanding retinopathy of prematurity: a Danish national study. Ophthalmology. 2016;123:796-803.
164. Sood V, Chellani H, Arya S, Guliani B. Changing spectrum of retinopathy of prematurity (ROP) and variations among siblings of multiple gestation. Indian J Pediatr. 2012;79:905-10.
165. Sood BG, Madan A, Saha S, Schendel D, Thorsen P, Skogstrand K, et al. Perinatal systemic inflammatory response syndrome and retinopathy of prematurity. Pediatr Res. 2010;67:394-400.
166. Reyes ZS, Al-Mulaabed SW, Bataclan F, Montemayor C, Ganesh A, Al-Zuhaibi S, et al. Retinopathy of prematurity: revisiting incidence and risk factors from Oman compared to other countries. Oman J Ophthalmol. 2017;10:26-32.
167. Stanković-Babić G, Oros A, Vujanović M, Cekić S, Jonović M. Some of the risk factors for retinopathy of prematurity. Acta Med Med. 2014;53:5-10.
168. Sveinsdóttir K, Ley D, Hövel H, Fellman V, Hüppi PS, Smith LE, et al. Relation of retinopathy of prematurity to brain volumes at term equivalent age and developmental outcome at 2 years of corrected age in very preterm infants. Neonatology. 2018;114:46-52.
169. Sun H, Dong Y, Liu Y, Chen Q, Wang Y, Cheng B, et al. Using ROPScore and CHOP ROP for early prediction of retinopathy of prematurity in a Chinese population. Ital J Pediatr. 2021;47:39.
170. Tadesse M, Dhanireddy R, Mittal M, Higgins RD. Race, Candida sepsis, and retinopathy of prematurity. Neonatology. 2002;81:86-90.

171. Taqui AM, Syed R, Chaudhry TA, Ahmad K, Salat MS. Retinopathy of prematurity: frequency and risk factors in a tertiary care hospital in Karachi, Pakistan. J Pak Med Assoc. 2008;58:186-90.
172. Teoh S, Boo N, Ong L, Nyein M, Lye M, Au M. Duration of oxygen therapy and exchange transfusion as risk factors associated with retinopathy of prematurity in very low birthweight infants. Eye (Lond). 1995;9:733-7.
173. Thomas K, Shah P, Canning R, Harrison A, Lee S, Dow K. Retinopathy of prematurity: risk factors and variability in Canadian neonatal intensive care units. J Neonatal Perinatal Med. 2015;8:207-14.
174. Tioseco JA, Aly H, Essers J, Patel K, El-Mohandes AA. Male sex and intraventricular hemorrhage. Pediatr Crit Care Med. 2006;7:40-4.
175. Todd D, Kennedy J, Roberts V, John E. Risk factors in progression beyond stage 2 retinopathy of prematurity. Aust N Z J Ophthalmol. 1990;18:57-60.
176. Todd D, Goyen T, Smith J, Rochefort M. Developmental outcome in preterm infants< 29 weeks gestation with ⩽ stage 3 retinopathy of prematurity (ROP): relationship to severity of ROP. J Dev Orig Health Dis. 2012;3:116-22.
177. Tolia VN, Ahmad KA, Jacob J, Kelleher AS, McLane N, Arnold RW, et al. Two-year outcomes of infants with stage 2 or higher retinopathy of prematurity: results from a large multicenter registry. American J Perinatol. 2020;37:196-203.
178. Tsui I, Ebani E, Rosenberg JB, Lin J, Angert RM, Mian U. Patent ductus arteriosus and indomethacin treatment as independent risk factors for plus disease in retinopathy of prematurity. J Pediatr Ophthalmol Strabismus. 2013;50:88-92.
179. Uchida A, Miwa M, Shinoda H, Koto T, Nagai N, Mochimaru H, et al. Association of maternal age to development and progression of retinopathy of prematurity in infants of gestational age under 33 weeks. J Ophthalmol. 2014;2014:187929.
180. Ueda K, Miki A, Nakai S, Yanagisawa S, Nomura K, Nakamura M. Prediction of severe retinopathy of prematurity using the weight gain, insulin-like growth factor 1, and neonatal retinopathy of prematurity algorithm in a Japanese population of preterm infants. Jpn J Ophthalmol. 2020;64:223-7.
181. Ugurbas SC, Gulcan H, Canan H, Ankarali H, Torer B, Akova YA. Comparison of UK and US screening criteria for detection of retinopathy of prematurity in a developing nation. J AAPOS. 2010;14:506-10.
182. Van der Merwe S, Freeman N, Bekker A, Smith J, Harvey J. Prevalence of and risk factors for retinopathy of prematurity in a cohort of preterm infants treated exclusively with non-invasive ventilation in the first week after birth. S Afr Med J. 2013;103:96-100.
183. van Sorge AJ, Termote JU, Kerkhoff FT, van Rijn LJ, Simonsz HJ, Peer PG, et al. Nationwide inventory of risk factors for retinopathy of prematurity in the Netherlands. J Pediatr. 2014;164:494-8.e1.
184. Vasavada D, Sengupta S, Prajapati VK, Patel S. Incidence and risk factors of retinopathy of prematurity in Western India–report from a regional institute of ophthalmology. Nepal J Ophthalmol. 2017;9:112-20.
185. Vucinovic M, Znaor L, Vucinovic A, Capkun V, Bandic J. Incidence and characteristics of infants with retinopathy of prematurity in Croatia. Int Ped Chi Care. 2018;1:35-45.
186. Wang ZH, Gao PF, Bai H, Li YY. Postnatal weight gain in very low birth weight infants in Beijing and the risk of retinopathy of prematurity. Int J Ophthalmol. 2015;8:1207-10.
187. Wang LW, Lin YC, Wang ST, Huang CC, on behalf of the Taiwan Premature Infant Developmental Collaborative Study Group. Identifying risk factors shared by bronchopulmonary dysplasia, severe retinopathy, and cystic periventricular leukomalacia in very preterm infants for targeted intervention. Neonatology. 2018;114:17-24.
188. Wang SK, Callaway NF, Wallenstein MB, Henderson MT, Leng T, Moshfeghi DM. SUNDROP: six years of screening for retinopathy of prematurity with telemedicine. Can J Ophthalmol. 2015;50:101-6.

189. Wani VB, Uboweja AK, Gani M, Al-Kandari J, Kazem M, Al-Naqeeb N, et al. Type I retinopathy of prematurity in infants with birth weight less than 1251 g: incidence and risk factors for its development in a nursery in Kuwait. Middle East Afr J Ophthalmol. 2013;20:66-71.
190. Wikstrand MH, Hård AL, Niklasson A, Smith L, Löfqvist C, Hellström A. Maternal and neonatal factors associated with poor early weight gain and later retinopathy of prematurity. Acta Paediatr. 2011;100:1528-33.
191. Woo SJ, Park KH, Jung HJ, nae Kim S, Choe G, Ahn J, et al. Effects of maternal and placental inflammation on retinopathy of prematurity. Graefes Arch Clin Exp Ophthalmol. 2012;250:915-23.
192. Wu T, Zhang L, Tong Y, Qu Y, Xia B, Mu D. Retinopathy of prematurity among very low-birth-weight infants in China: incidence and perinatal risk factors. Invest Ophthalmol Vis Sci. 2018;59:757-63.
193. Yang CY, Lien R, Yang PH, Chu SM, Hsu JF, Fu RH, et al. Analysis of incidence and risk factors of retinopathy of prematurity among very-low-birth-weight infants in North Taiwan of China. Pediatr Neonatol. 2011;52:321-6.
194. Yang X, Ze B, Dai Y, Zhu L, Chen C. The relationship between cytokines serum levels at postnatal 4-6 weeks and retinopathy of prematurity. HK J Paediatr (New Series). 2017;22:3-9.
195. Yang MB. Retinopathy of prematurity in infants with birth weight ≥ 1250 grams—incidence, severity, and screening guideline cost-analysis. J AAPOS. 2007;11:208-10.
196. Yanovitch TL, Siatkowski RM, McCaffree M, Corff KE. Retinopathy of prematurity in infants with birth weight ≥ 1250 grams—incidence, severity, and screening guideline cost-analysis. J AAPOS. 2006;10:128-34.
197. Yau GS, Lee JW, Tam VT, Liu CC, Chu BC, Yuen CY. Incidence and risk factors for retinopathy of prematurity in extreme low birth weight Chinese infants. Int Ophthalmol. 2015;35:365-73.
198. Ying GS, Bell EF, Donohue P, Tomlinson LA, Binenbaum G, G-ROP Research Group. Perinatal risk factors for the retinopathy of prematurity in postnatal growth and rop study. Ophthalmic Epidemiol. 2019;26:270-8.
199. Yu XD, Branch DW, Karumanchi SA, Zhang J. Preeclampsia and retinopathy of prematurity in preterm births. Pediatrics. 2012;130:e101-7.
200. Yu Y, Tomlinson LA, Binenbaum G, Ying G, G-Rop Study Group. Incidence, timing and risk factors of type 1 retinopathy of prematurity in a North American cohort. Br J Ophthalmol. 2021;105:1724-30.
201. Zarei M, Bazvand F, Ebrahimiadib N, Roohipoor R, Karkhaneh R, Farahani Dastjani A, et al. Prevalence and risk factors of retinopathy of prematurity in Iran. J Ophthalmic Vis Res. 2019;14:291-8.
202. Zepeda-Romero LC, Padilla-Muñoz H, Miguel AJÓ, Ricardo GS, Plascencia-Alonso M, Peña-Pérez ML, et al. Risk factors in retinopathy of prematurity in UCINEX in the Civil Hospital of Guadalajara. Rev Med MD. 2014;5:189-94.
203. Zisk JL, Genen LH, Kirkby S, Webb D, Greenspan J, Dysart K. Do premature female infants really do better than their male counterparts? Am J Perinatol. 2011;28:241-6.
